# Benchmarking community drug response prediction models: datasets, models, tools, and metrics for cross-dataset generalization analysis

**DOI:** 10.1093/bib/bbaf667

**Published:** 2026-01-12

**Authors:** Alexander Partin, Priyanka Vasanthakumari, Oleksandr Narykov, Andreas Wilke, Natasha Koussa, Sara E Jones, Yitan Zhu, Jamie C Overbeek, Rajeev Jain, Gayara Demini Fernando, Cesar Sanchez-Villalobos, Cristina Garcia-Cardona, Jamaludin Mohd-Yusof, Nicholas Chia, Justin M Wozniak, Souparno Ghosh, Ranadip Pal, Thomas S Brettin, M Ryan Weil, Rick L Stevens

**Affiliations:** Computing, Environment and Life Sciences, Argonne National Laboratory, 9700 S Cass Ave, Lemont, 60439 IL, United States; Computing, Environment and Life Sciences, Argonne National Laboratory, 9700 S Cass Ave, Lemont, 60439 IL, United States; Computing, Environment and Life Sciences, Argonne National Laboratory, 9700 S Cass Ave, Lemont, 60439 IL, United States; Computing, Environment and Life Sciences, Argonne National Laboratory, 9700 S Cass Ave, Lemont, 60439 IL, United States; Cancer Data Science Initiatives, Cancer Research Technology Program, Frederick National Laboratory for Cancer Research, 8560 Progress Drive, Frederick, 21701 MD, United States; Cancer Data Science Initiatives, Cancer Research Technology Program, Frederick National Laboratory for Cancer Research, 8560 Progress Drive, Frederick, 21701 MD, United States; Computing, Environment and Life Sciences, Argonne National Laboratory, 9700 S Cass Ave, Lemont, 60439 IL, United States; Computing, Environment and Life Sciences, Argonne National Laboratory, 9700 S Cass Ave, Lemont, 60439 IL, United States; Computing, Environment and Life Sciences, Argonne National Laboratory, 9700 S Cass Ave, Lemont, 60439 IL, United States; Department of Statistics, University of Nebraska-Lincoln, 3310 Holdrege St, Lincoln, 68583 NE, United States; Department of Electrical and Computer Engineering, Texas Tech University, 910 Boston Ave, Lubbock, 79409 TX, United States; Division of Computer, Computational and Statistical Sciences, Los Alamos National Laboratory, Los Alamos, 87545 NM, United States; Division of Computer, Computational and Statistical Sciences, Los Alamos National Laboratory, Los Alamos, 87545 NM, United States; Computing, Environment and Life Sciences, Argonne National Laboratory, 9700 S Cass Ave, Lemont, 60439 IL, United States; Computing, Environment and Life Sciences, Argonne National Laboratory, 9700 S Cass Ave, Lemont, 60439 IL, United States; Department of Statistics, University of Nebraska-Lincoln, 3310 Holdrege St, Lincoln, 68583 NE, United States; Department of Electrical and Computer Engineering, Texas Tech University, 910 Boston Ave, Lubbock, 79409 TX, United States; Computing, Environment and Life Sciences, Argonne National Laboratory, 9700 S Cass Ave, Lemont, 60439 IL, United States; Cancer Data Science Initiatives, Cancer Research Technology Program, Frederick National Laboratory for Cancer Research, 8560 Progress Drive, Frederick, 21701 MD, United States; Computing, Environment and Life Sciences, Argonne National Laboratory, 9700 S Cass Ave, Lemont, 60439 IL, United States; Department of Computer Science, The University of Chicago, 5730 S Ellis Ave, Chicago, 60637 IL, United States

**Keywords:** drug response prediction, model benchmarking, cross-dataset generalization, cross-study analysis, precision oncology, deep learning

## Abstract

Deep learning and machine learning models have shown promise in drug response prediction (DRP), yet their ability to generalize across datasets remains an open question, raising concerns about their real-world applicability. Due to the lack of standardized benchmarking approaches, model evaluations and comparisons often rely on inconsistent datasets and evaluation criteria, making it difficult to assess true predictive capabilities. In this work, we introduce a benchmarking framework for evaluating cross-dataset prediction generalization in DRP models. Our framework incorporates five publicly available drug screening datasets, seven standardized DRP models, and a scalable workflow for systematic evaluation. To assess model generalization, we introduce a set of evaluation metrics that quantify both absolute performance (e.g. predictive accuracy across datasets) and relative performance (e.g. performance drop compared to within-dataset results), enabling a more comprehensive assessment of model transferability. Our results reveal substantial performance drops when models are tested on unseen datasets, underscoring the importance of rigorous generalization assessments. While several models demonstrate relatively strong cross-dataset generalization, no single model consistently outperforms across all datasets. Furthermore, we identify CTRPv2 as the most effective source dataset for training, yielding higher generalization scores across target datasets. By sharing this standardized evaluation framework with the community, our study aims to establish a rigorous foundation for model comparison, and accelerate the development of robust DRP models for real-world applications.

## Introduction

Predictive modeling has become an integral tool in cancer research, offering new capabilities for improving clinical outcomes [1, 2]. Anticancer drug response prediction (DRP) models provide an *in silico* approach to evaluating the potential effects of drugs on cancer, leveraging artificial intelligence (AI) techniques such as deep learning (DL) and classical machine learning (ML) [3–5]. The increasing availability of high-throughput genomic profiling and drug screening data has enabled the rapid development of these models. However, despite the growing number of DRP models, it remains unclear how well their predictive performance translates to unseen datasets and experimental conditions, limiting their potential for broader real-world applicability [1, 3, 6].

While the ultimate goal is to accurately predict drug responses in patients, *in vitro* cell line drug screenings remain the most abundant source of data and, therefore, the primary resource for developing and evaluating DRP models. However, while many published models achieve high predictive accuracy within a single screening dataset, their performance deteriorates when applied to more complex biological systems such as organoids, patient-derived xenografts (PDX), or patients samples [7, 8]. Therefore, an intermediate step in complexity involves assessing prediction generalization across different cell line datasets, a practice that has recently become widely common [6]. Demonstrating robust cross-dataset generalization goes beyond simple cross-validation within a single dataset and positions a model as a promising candidate for more complex transfer tasks.

Despite the growing number of DRP models, the lack of standardized frameworks for scalable benchmarking remains a major challenge in identifying effective methods for cancer treatment prediction. Performance evaluation is often conducted with simple baselines, varied dataset compositions, inconsistent data splits, and diverse scoring metrics, impeding assessments of model strengths, weaknesses, and key factors contributing to model performance. To demonstrate tangible progress in the field and better understand model capabilities, DRP models should undergo systematic cross-comparisons against multiple other models, ideally demonstrating sufficient cross-dataset generalization, in a rigorous and systematic way. Collaborative community efforts have emerged to establish guidelines and best practices for improving model development and evaluation [1, 3, 6]. Recent evaluation studies conducted model comparisons, focusing on various aspects including cross-dataset prediction [6, 9, 10], but often lack public tools or guidelines for expanding the analysis to more datasets, models, or evaluation schemes (see Supplementary Table S1 for detailed comparison).

To address the pressing need for standardized benchmarking in DRP, this study, conducted as part of IMPROVE [11, 12] project, aims to establish a framework for systematic evaluation and comparison of DL models, applicable to various scientific domains. We present simple benchmarking principles centered around four key aspects: (i) benchmark datasets, (ii) standardized models, (iii) software tools and protocols, and (iv) evaluation workflows. These principles, which form the core contributions of this study, are designed to facilitate systematic model benchmarking. Specifically, we constructed a comprehensive benchmark dataset (Section Benchmark dataset) comprising drug response data from five publicly available drug screening studies (Table 1), along with drug and omics feature representations and precomputed data splits to ensure consistency across evaluations. We also designed a unified code structure promoting modular design and developed *improvelib*, a lightweight Python package that standardizes preprocessing, training, and evaluation, ensuring consistent model execution and enhancing reproducibility. Furthermore, we selected five DL-based DRP models and one ML model built with LightGBM [13] (Table 2) and adjusted their code to conform to the required modular structure. Finally, we implemented a scalable workflow to conduct large-scale cross-dataset analysis, applying it to the selected models and benchmark dataset for comprehensive training and evaluation.

**Table 1:** Summary of the number of unique drugs, cell lines, and AUC response across drug screening datasets.

Dataset	Drugs	Cell lines	Responses
CCLE	24	411	9519
CTRPv2	494	720	286 665
gCSI	16	312	4941
GDSCv1	294	546	171 940
GDSCv2	168	470	114 644

**Table 2:** Overview of seven benchmarked drug response prediction models, including computational frameworks and the cancer and drug feature types used.

	Model	Year	Framework	Cancer omics features	Drug features	Architecture components
1	DeepCDR [30]	2020	TF-Keras	GE, Methyl, Mu	MG	Batchnorm, Dropout
2	DeepTTC [31]	2022	PyTorch	GE	FPs	Transformer (drugs), MLP (GE)
3	GraphDRP [32]	2022	PyTorch	CNV, Mu	MG	Batchnorm, Dropout, GIN (drugs), 1D-CNN (GE)
4	HiDRA [33]	2021	TF-Keras	GE	FPs	Attention, Batchnorm
5	tCNNs [19]	2019	TF	CNV, Mu	SMILES (one-hot)	1D-CNN (CNV, Mu, SMILES), Dropout
6	UNO [9]	2022	TF-Keras	GE	DD, FPs	Dropout, RC
7	LGBM		LightGBM	GE	DD	

Abbreviations: TF, TensorFlow; GE, gene expression; Methyl, methylation; Mu, mutation; CNV, copy number variation; DD, drug descriptors; MG, molecular graph; FPs, fingerprints; SMILES, simplified molecular-input line-entry system; GIN, graph isomorphism network; 1D-CNN, one-dimensional convolutional neural network; RC, residual connections.

## Materials and methods

**Figure 1:**
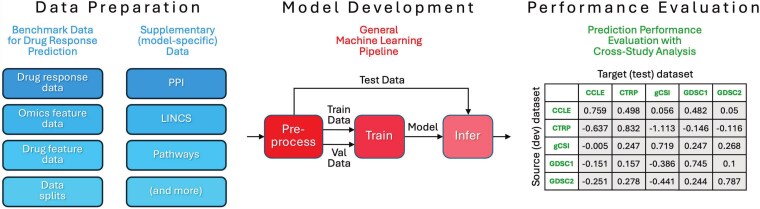
Basic components in the development of DRP models, comprising (i) data preparation: including benchmark data components (e.g. drug response, omics, and drug feature data) and supplementary model-specific data; (ii) model development: a general ML pipeline structured into three distinct stages—preprocessing, training, and inference; and (iii) performance evaluation: cross-dataset generalization assessment (the example $G$ matrix is shown for illustration purposes and does not represent real experimental results).

The development of DRP models, much like any supervised learning model, typically includes three core components: data preparation, model development, and performance analysis [3]. The specifics of each component are often contingent upon the application, performance evaluation scheme, required rigor, and scale of the analysis. Within this general framework, we outline the details of the analysis conducted in this work (Fig. 1).

A benchmark dataset, the product of the data preparation step, is essential for fair analysis and a required ingredient for establishing state-of-the-art among models. In the context of our analysis, which investigates DRP models, this dataset comprises drug response data compiled from multiple drug screening studies, omics and drug feature data, and precomputed data splits to ensure consistent train, validation, and test sets.

Model development, in this context, refers to an ML learning pipeline comprising data preprocessing, model training, and inference. These steps take the output of data preparation (benchmark data and potentially additional model-specific data), transform it into suitable model input data, train the model, and generate predictions on a hold-out set through inference.

**Figure 2:**
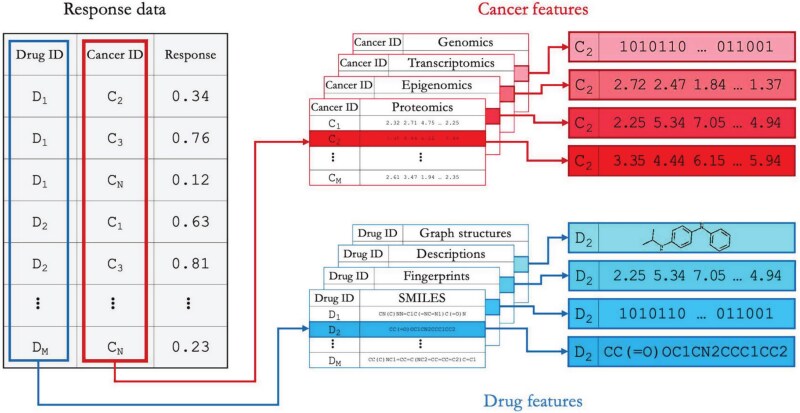
The main data components in the drug response benchmark dataset and their integration through shared cell and drug identifiers.

Finally, the evaluation scheme can encompass a wide array of approaches, ranging from basic cross-validation or hold-out set analysis on a single dataset to more complex schemes. These may include cross-dataset generalization across studies from the same [9, 14, 15] or different biological media [7, 16–18], data scaling analysis via learning curves [9, 19–21], and interpretability assessments [10, 22]. In this work, a workflow for conducting scalable cross-dataset generalization analysis with the benchmark dataset and seven DRP models is presented.

The rest of the Materials and methods section details our approach, demonstrated with seven prediction models and the benchmark dataset. We describe the benchmark dataset’s characteristics (Section Benchmark dataset), the models and the model selection criteria (Section Models), the methodology for standardizing models to ensure consistent evaluation (Section Standardizing models), and the cross-dataset evaluation scheme used (Section Cross-dataset workflow).

### Benchmark dataset

The benchmark dataset consists of three main components: response data, cancer features, and drug features. These components include data types commonly used by pan-cancer and multi-drug models for predicting treatment response [3]. Unique cancer and drug identifiers link the components, allowing integration into structured formats for model training and evaluation.

#### Drug response data

The drug response data were gathered from five public drug screening studies: Cancer Cell Line Encyclopedia (CCLE) [23], Cancer Therapeutics Response Portal (CTRPv2) [24], Genentech Cell Line Screening Initiative (gCSI) [25], and Genomics of Drug Sensitivity in Cancer project, which includes GDSCv1 and GDSCv2 datasets [26]. The response in cell lines was quantified by measuring cell viability across multiple drug doses. We fitted each cell–drug pair’s dose–response data to a three-parameter Hill–Slope curve, excluding pairs with $ R^{2} $ ¡ 0.3 to ensure data quality. The area under the curve (AUC) was calculated over a dose range of $[10^{-10}$M, $10^{-4}$M$]$ and normalized to [0, 1], with lower AUC values indicating stronger response (higher growth inhibition). Each cell–drug pair AUC value represents a single drug response sample. Table 1 summarizes the number of unique drugs, cell lines, and total AUC responses in each dataset, constituting the benchmark data.

#### Multiomics data

The multiomics data used for cell line representation were sourced from the Dependency Map (DepMap) portal of CCLE version 22Q2 (https://depmap.org/portal) [27]. This dataset includes gene expressions, DNA mutations, DNA methylation, gene copy numbers, protein expressions (RPPA), and miRNA expressions. The gene expression data, derived from RNA-seq and log2-transformed with a pseudo count of 1, covers 1007 cell lines across 30 805 genes. The mutation data in long format provide details on somatic point mutations for 1024 cell lines, including the mutated gene, variant classification, genome change, protein change, and chromosomal positions. Additionally, two derivative datasets were curated: binary mutation data, which identifies the presence of mutations in a gene within a cell line, and mutation count data, representing the number of mutations per gene in each cell line. The DNA methylation data contain methylation levels at transcription start sites for 824 cell lines. The copy number data include log2-transformed gene-level copy number values for 1018 cell lines across 25 331 genes. The RPPA data report protein expression levels for 789 cell lines mapped to 214 target genes through antibody-based proteomics. The miRNA data include expression levels for 820 cell lines across 734 miRNAs.

#### Drug representation data

In addition to drug response and omics data, the benchmark includes drug metadata and three types of drug representations: SMILES, fingerprints (FPs), and descriptors. Although SMILES are rarely used in their raw string format in DRP models, they remain a popular notation for describing molecules. SMILES often serve as an intermediate step for generating other representations, such as fingerprints, molecular descriptors, and graph structures. Both raw and canonicalized SMILES are provided in the dataset. FPs and molecular descriptors are perhaps the two most common feature types for representing drugs in DRP papers [3]. Unlike SMILES, where string length varies for different molecules, FPs and descriptors allow for a consistent number of features across all drugs in a dataset. This fixed feature dimensionality makes them easier to use with prediction models. We used the open-source package RDKit [28] to generate 512-bit Extended Connectivity Fingerprints via the Morgan algorithm. Descriptors were calculated using the open-source Mordred package [29]. The full descriptor set comprises 1826 continuous and discrete features, including both 2D and 3D molecular structure descriptors. Since most 3D descriptors resulted in invalid (NaN) values for the majority of compounds, we retained only the 2D descriptors, providing a total of 1613 drug features.

#### Data partitions

The final component of the benchmark data comprises multiple sets of disjoint data splits, used to ensure consistent training, validation, and test data utilized across all models, thereby enhancing the rigor of the analysis. For each of the five datasets in Table 1, we generated 10 data splits using random 10-fold cross-validation, resulting in 10 sets of training ($T$), validation ($V$), and test ($E$) samples per dataset, represented as $\{T, V, E\}_{n=1}^{N}$ where $N=10$. Each set $\{T, V, E\}$ maintains a size proportion of (0.8, 0.1, 0.1) relative to the total number of drug response samples in a dataset. The splits are stored as text files containing row indices corresponding to the drug response data, allowing for easy retrieval of specific subsets.

### Models

We previously compiled a comprehensive list of >60 papers (as of August 2022) proposing DL-based models for monotherapy DRP [3]. From this list, we selected 17 models for a reusability and reproducibility study, assessing the ease of reusing and adapting these models to new datasets and contexts [12]. In the current study, we further refined this selection to seven models from the reusability study and included one additional model, called UNO [9]. Section “Model selection process” outlines the primary selection criteria used in [12] that remain applicable here, along with additional criteria leading to the final selection of models listed in Table 2.

#### Model selection process

We selected a subset of models based on qualitative and empirical criteria.

Qualitative criteria:

1. Model is accompanied by open-source code, with preference given to publications from 2019 onward
2. DL model implemented in PyTorch, TensorFlow, and Keras
3. Model predicts monotherapy drug response values using an end-to-end learning approach
4. Code repository includes clear instructions on how to use the model and reproduce results
5. Pan-cancer and pan-drug model (i.e. learns from cancer and drug representations)

Empirical criteria:

1. We successfully installed the computational environment
2. We successfully executed preprocessing scripts and generate model-input data
3. We were able to reproduce key results reported in the original publications

#### Selected models

Following the selection process outlined in Section “Model selection process,” a total of five DL models were selected for this study together with LGBM, a LightGBM-based model. Table 2 lists these models, including the required omics and drug representations, and the unique neural network building blocks employed in each model’s architecture. The selected models demonstrate a range of design approaches, encompassing single- to multi-modal architectures and incorporating diverse neural network components such as convolutional neural networks, graph neural networks (GNNs), and attention mechanisms.

### Standardizing models

Computational workflows can be designed to conduct systematic and rigorous analyses across various models and experimental conditions. The inconsistent code structures of different models hinder the straightforward design of such workflows. We address this by standardizing models through several key principles. An essential prerequisite for designing workflows is that the models’ code should be structured in a modular fashion, allowing easy, upon-request invocation of specific modules consistently across models. This approach enables workflow developers to design workflows without requiring in-depth knowledge of specific model details.

**Figure 3:**
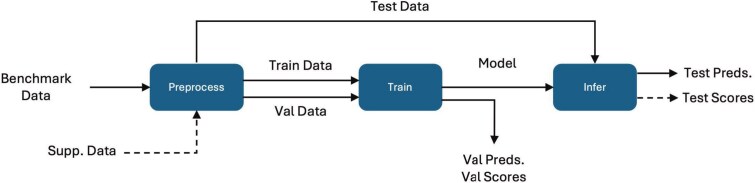
General ML pipeline illustrating preprocessing, training, and inference, with dashed lines denoting optional elements, including supplementary data which refers to model-specific data not part of the benchmark dataset (e.g. KEGG pathway gene set from the MSigDB [34, 35] used by HiDRA [33]).

The first principle involves standardized code structure. Prediction models, regardless of the application, generally consist of three main stages in the ML pipeline (see Fig. 3): (i) **preprocessing** to transform benchmark data into model-input data, (ii) **training** to obtain a prediction model, and (iii) **inference** to compute predictions. We require the separation of these stages into distinct model scripts, allowing flexible workflow design where each stage can be called as a module.

Second, we enforce a unified interface for interacting with these scripts, ensuring consistent inputs and outputs. To create a generalizable design applicable across various scientific domains, the interface supports three sets of input parameters for each model script: (i) general *improvelib* parameters, (ii) application-specific parameters (where *application* refers to a specific use case such as DRP), and model-specific parameters. This tiered parameter structure enhances flexibility and generalizability across diverse applications and models.

The third component, crucial for achieving model standardization, involves a set of utility tools incorporated in the *improvelib* Python package. This lightweight package, with minimal standard dependencies, provides the necessary functionalities to support the three model stage scripts and the unified interface. It enables proper parameter precedence (command line, config file, and defaults), generates user-friendly help messages, and facilitates logging of experimental conditions.

Figure 3 demonstrates the general ML pipeline, encompassing preprocessing, training, and inference stages. Table 3 details the inputs, outputs, and key functionalities provided by *improvelib* for each ML stage, ensuring consistently across all standardized models.

**Table 3:** Primary inputs, outputs, and functionality of preprocessing, training, and inference scripts, with further details on model standardization and practical examples provided in Supplementary Material S7.

	Inputs	Outputs	*improvelib* functionality
Preprocessing	Benchmark data	Train data	*DRPPreprocessConfig*
	Supplementary data	Val data	*OmicsLoader, DrugsLoader,*
		Test data	*DrugResponseLoader*
Training	Train data	Val data predictions	*DRPTrainConfig,*
	Val data	Val data performance scores	*store_predictions_df,*
		Trained model	*compute_performance:scores*
Inference	Test data	Test data predictions	*DRPInferConfig*
	Trained model	Test data performance scores (optional)	*store_predictions_df*
			*compute_performance:scores*

**Preprocessing.** The preprocessing script handles benchmark data for all models evaluated. The Python package includes designated data loaders for various benchmark files, including cancer and drug feature data ($x$ data), drug response data ($y$ data), and data splits. All models are expected to use these loaders to load benchmark data files. Some models utilize additional data types beyond what is included in the benchmark data. For example, HiDRA incorporates gene set data from KEGG pathways, sourced from the Molecular Signatures Database (MSigDB) [34, 35] to structure gene expression features into pathway-level representations [33]. The preprocessing interface accommodates this via an optional input argument specifying the path to model-specific data (see Supplementary Material S2 for HiDRA’s implementation details). The preprocessing script then uses this path to load the additional data and integrates it with the benchmark data as required by the model. The output of this stage consists of three model-ready datasets: training, validation, and test data, each formatted specifically for the model’s framework (e.g. *.pt* for PyTorch, *.tfrecords* for TensorFlow).

**Training**. The training script uses the preprocessed training and validation data to produce a trained model, along with raw predictions and performance scores for the validation set. To ensure convergence, all models implement early-stopping and use validation data to terminate training if validation loss does not improve for a predefined number of iterations (the model with the lowest validation loss is saved for the inference stage). The package includes standardized methods for saving the trained model to a predefined path and store predictions and scores with consistent naming conventions. This simplifies model loading during inference and facilitates result aggregation in post-processing.

**Inference**. Finally, the inference script takes the trained model and applies it to the preprocessed test data, generating and saving test set predictions and performance scores. Similar to the validation process, test predictions and scores are stored in consistently named output files, which are then aggregated in post-processing script to produce the final results.

### Cross-dataset workflow

In our prior works [9, 36], we conducted cross-study analysis to evaluate prediction generalization of models across multiple drug screening studies. However, those analyses were limited to a smaller set of models and were not designed as a systematic benchmarking framework. In this work, we extend that methodology and adopt the broader term *cross-dataset generalization*, which reflects its applicability beyond biological studies.

The cross-dataset evaluation scheme assesses prediction generalization both across and within datasets. The *source* dataset is the drug response data from a drug screening dataset used for model development, including training and validation data. The *target* dataset is a separate set of drug response samples used exclusively for model evaluation, which may either be from the same dataset (within-dataset evaluation) or a different dataset (cross-dataset evaluation).

We utilized $ d = 5 $ datasets (listed in Table 1), resulting in a $ d $-by-$ d $ matrix $ G $ for each model. For each source dataset $ s $, we generated $ N=10 $ train/validation/test splits and trained one model instance per split, resulting in 10 trained models per source. These trained models were then evaluated on all $ d $ target datasets $ t $. For within-dataset performance ($ s=t $), each model instance is evaluated on the corresponding test split. For cross-dataset performance ($ s \neq t $), each model instance is evaluated on the full target dataset. The final entry $ g[s,t] $ is computed as the average score across the 10 model instances, as described in Section “Performance metrics.” This results in 50 total trained model instances per architecture (10 per dataset), which are reused across target datasets for predictions.

Hyperparameter selection was limited to batch size, learning rate, and dropout rate (for models incorporating dropout layers). These values were manually adjusted based on the original model publications but were not subjected to exhaustive tuning. This approach ensured that training configurations remained aligned with prior studies while allowing for stable performance across datasets.

**Figure 4:**
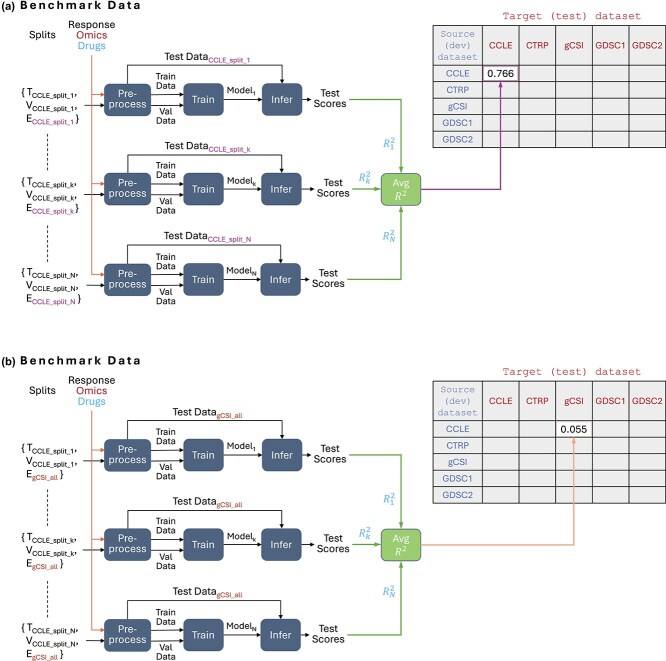
This figure illustrates cross-dataset generalization by showing the computation of the prediction performance score for the $ G[CCLE, CCLE] $ matrix entry (within-study, a) and the $ G[CCLE, gCSI] $ entry (cross-dataset, b), with the workflow executed in parallel using Parsl.

Figure 4A and B illustrates the workflows for generating within-dataset and cross-dataset results, respectively. In Fig. 4A, training, validation, and test data originate from the same dataset (e.g. CCLE split 0), and the average score $ g[s,s] $ is assigned to the diagonal entry in $ G $. In Fig. 4B, training and validation data are from one dataset (e.g. CCLE split 0), while the test data are from a different dataset (e.g. gCSI). The averaged score $ g[s,t] $ is then assigned to the corresponding off-diagonal entry in $ G $.

It is important to note that although the response values ($ y $) in the target dataset remain constant across ML pipeline runs in the case of Fig. 4B, the feature values ($ x $) may vary due to preprocessing steps like feature scaling. This preprocessing, often dependent on the training data, may introduce variation in the feature values of the target dataset across different pipeline runs.

As shown in Fig. 4, the ML pipelines for $ N $ splits can be executed in parallel. To facilitate parallel execution, we implemented this workflow using the Parsl parallel processing library [37]. Each pipeline component—preprocessing, training, and inference—was designed as a Parsl app, returning a *futures* object to monitor execution progress. For instance, once preprocessing for CCLE split 1 is complete, the corresponding *futures* object triggers training for that split. Similarly, inference is initiated upon training completion, with its own *futures* object. This parallelized workflow was executed for all models on an 8-GPU cluster at Argonne National Laboratory.

### Performance metrics

In our previous work [9], we analyzed cross-dataset results by presenting raw prediction performance scores in a $ d $-by-$ d $ matrix, where $ d $ represents the number of datasets. Here, we extend that framework by introducing four key metrics: $ G $, $ G_{a} $, $ G_{n} $, and $ G_{na} $. These metrics provide deeper insights into the generalization capabilities of models by capturing both absolute cross-dataset performance and performance relative to the observed within-dataset performance.

#### Basic cross-dataset performance matrix (G)

**Figure 5:**
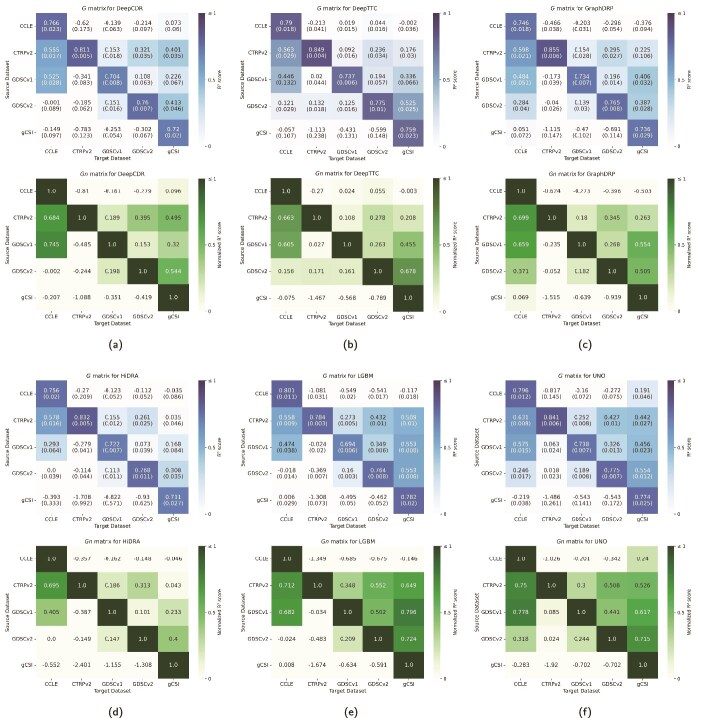
Cross-dataset performance matrices for each model, with each subfigure displaying the basic matrix $ G $ (blue) and the normalized matrix $ G_{n} $ (green), with mean $ R^{2} $ scores and standard deviations across splits (the heatmaps for tCNNS is shown in Supplementary Fig. S6 to preserve space and improve clarity.

The matrix $ G $ represents a model’s performance trained on a source dataset $ s $ and evaluated on a target dataset $ t $, where $ s, t \in \{1, 2, \dots , d\} $ are datasets indices. Each entry $ g[s,t] $ in $ G $ is the average prediction generalization score across $ N $ independent data splits:


\begin{align*} & g[s,t] = \frac{1}{N} \sum_{n=1}^{N} g[s,t,n], \quad \forall \, s, t \in \{1, 2, \dots, d\} \end{align*}

where $ g[s,t,n] $ is the coefficient of determination ($ R^{2} $) for the $ n $th split of the model trained on $ s $ and evaluated on $ t $. We selected $ R^{2} $ as a standard regression metric in DRP to measure the proportion of variance in drug response explained by the model, enabling consistent cross-dataset comparisons [9]. Performance is reported as the mean $ R^{2} $ and standard deviation across splits, as shown in Fig. 5.

In our previous work [9], the $ G $ matrix was introduced to illustrate cross-dataset performance, though without a structured benchmarking framework. Here, we formally establish it as part of a systematic evaluation methodology. In the current study, we expand on this concept by ensuring consistent data splits across all evaluated models. Additionally, we report both the mean and standard deviation of performance scores for each entry $ g[s,t] $, providing a more comprehensive view of cross-dataset variability (Fig. 5).

#### Aggregated cross-dataset performance ($G_{a}$)

The metric $ G_{a} $ aggregates the entries of $ G $ across all target datasets, excluding the within-dataset evaluation ($ s = t $), to summarize the cross-dataset performance of a model trained on a given source dataset:


\begin{align*} & g_{a}[s] = \frac{1}{d-1} \sum_{\substack{t=1 \\ t \neq s}}^{d} g[s,t], \quad \forall \, s \in \{1, 2, \dots, d\} \end{align*}

where $ g[s,t] $ is an entry in $ G $. This aggregation provides a single summary value per source dataset, indicating how well a model trained on $ s $ generalizes on average to all other datasets. It highlights the intrinsic generalization capability of a source dataset without being influenced by within-dataset performance.

#### Normalized cross-dataset performance matrix ($G_{n}$)

The normalized matrix $ G_{n} $ extends $ G $ by scaling each entry $ g[s,t] $ by the corresponding within-dataset performance $ g[s,s] $. This normalization provides a relative measure of generalization:


\begin{align*} & g_{n}[s,t] = \frac{g[s,t]}{g[s,s]}, \quad \forall \, s, t \in \{1, 2, \dots, d\} \end{align*}

The normalized performance matrix $ G_{n} $ contextualizes each model’s performance on a target dataset $ g[s,t] $ relative to its baseline within-dataset performance $ g[s,s] $. This scaling quantifies the degree to which models trained on a source dataset $ s $ generalize to target dataset $ t $, highlighting whether their performance on $ t $ approaches or maintains the level observed within $ s $ itself.

#### Aggregated normalized cross-dataset performance ($G_{na}$)

The metric $ G_{na} $ aggregates the normalized performance scores $ g_{n}[s,t] $ across all target datasets (excluding $ s = t $) to summarize cross-dataset generalization relative to within-dataset performance:


\begin{align*} & g_{na}[s] = \frac{1}{d-1} \sum_{\substack{t=1 \\ t \neq s}}^{d} g_{n}[s,t], \quad \forall \, s \in \{1, 2, \dots, d\} \end{align*}

This metric provides a single summary score per source dataset $ s $, emphasizing how well a model generalizes across other datasets while accounting for its within-dataset performance.

These metrics collectively offer a comprehensive framework for evaluating cross-dataset performance. While $ G $ and $ G_{a} $ focus on absolute cross-dataset performance, $ G_{n} $ and $ G_{na} $ emphasize relative performance normalized by within-dataset results. Together, they provide insights into both pairwise dataset relationships and dataset-level generalization trends, offering tools to assess the broader applicability and robustness of models.

## Results

Despite leveraging advanced and diverse DL techniques (Table 2), all models demonstrate substantially lower performance in cross-dataset analysis compared with cross-validation within a single dataset (Fig. 5). This disparity underscores the inherent difficulty of achieving cross-dataset generalization in DRP and highlights the need for rigorous and systematic evaluation frameworks that closely reflect the complexities of real-world applications.

We use $ R^{2} $ which quantifies the proportion of variance in drug response explained by the model, making it a standard metric for DRP models predicting continuous outcomes like $ IC_{50} $ or AUC (area under the dose–response curve). Because $ R^{2} $ scales predictions by the variability of the observed data, it enables direct comparison of models across datasets, irrespective of scale differences. $ R^{2} $ ranges from 1 (perfect prediction) to negative values, where $ R^{2} = 0 $ indicates performance equivalent to a naive mean predictor (always predicting the mean response), and a negative $ R^{2} $ signifies worse performance than the naive mean predictor. This range of $ R^{2} $ is particularly informative in cross-dataset analysis, highlighting a model’s inability to generalize, as shown in prior work [9], where low to negative $ R^{2} $ revealed performance degradation across datasets. While alternative metrics like mean absolute error or Pearson correlation coefficient could complement $ R^{2} $, we use $ R^{2} $ for its interpretability in measuring explained variance and its clear indication of model failure through negative values.

### Within-dataset performance

The within-dataset evaluation measures a model’s predictive performance when training and testing are conducted on the same dataset. This analysis provides key insights into model performance and robustness under controlled conditions. Supplementary Table S3.1 and S3.2 summarize the mean and standard deviation of $ R^{2} $ scores across splits, respectively, reflecting on performance generalization and stability of these models.

Among the models, UNO achieves the highest mean $ R^{2} $ computed across datasets ($ 0.785 $), closely followed by GraphDRP ($ 0.767 $), LGBM ($ 0.765 $), HiDRA ($ 0.758 $), and DeepCDR ($ 0.752 $) (see SupplementaryTable S3.1 and Fig. 7). While UNO and GraphDRP perform slightly better than other models on the largest dataset, CTRPv2, differences in predictive accuracy between these top models are modest, and their performance ranges overlap when standard deviations are considered. LGBM, the only non-DL model in this analysis, achieves slightly higher predictive accuracy on smaller datasets (e.g. gCSI and CCLE), although the differences from DL models are not substantial. Conversely, tCNNS clearly exhibits lower mean accuracy ($ 0.632 $) and higher variability, distinguishing it from the other models.

**Figure 6:**
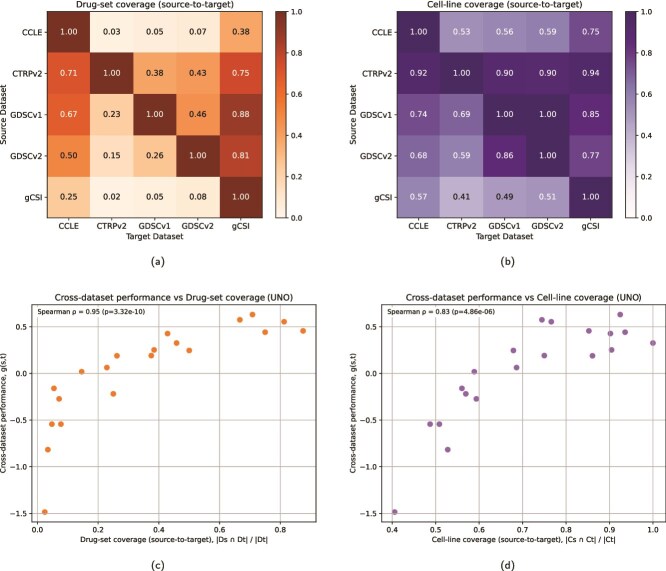
Coverage and cross-dataset performance shown in: (a) heatmap of *drug-set coverage* (source-to-target), with entries $C_{D}(s,t)=|D_{s} \cap D_{t}|/|D_{t}|$, where $D_{s}$ and $D_{t}$ are the unique compounds in source $s$ and target $t$, (b) heatmap of *cell-line coverage* (source-to-target), with entries $C_{C}(s,t)=|C_{s} \cap C_{t}|/|C_{t}|$, where $C_{s}$ and $C_{t}$ are the unique cell lines in source $s$ and target $t$, (c) scatter plot of cross-dataset performance $g(s,t)$ (off-diagonal pairs in a $G$ matrix for UNO) versus drug-set coverage $C_{D}(s,t)$, (d) scatter plot of $g(s,t)$ (off-diagonal pairs in a $G$ matrix for UNO) versus cell-line coverage $C_{C}(s,t)$.

**Figure 7:**
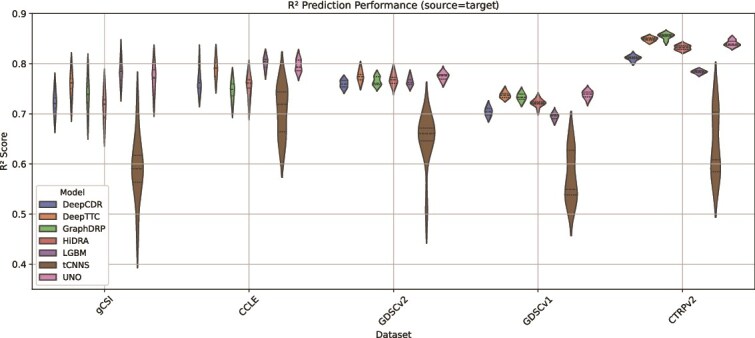
Violin plot showing $ R^{2} $ performance scores across models, datasets, and data splits in the within-dataset analysis, where each violin represents the approximate probability density of the data with the width indicating frequency of data points at each $ R^{2} $ value and markers indicating the median and interquartile range (datasets arranged by total sample size representing the number of cell–drug response samples).

Model stability, measured by the standard deviation of $ R^{2} $ scores across splits, reveals notable differences among models. LGBM demonstrates the lowest variability ($ 0.0096 $), reflecting stable performance across splits (see Supplementary Table S3.2). It is followed by UNO, DeepCDR, GraphDRP, and HiDRA. In contrast, tCNNS exhibits the highest variability ($ 0.0548 $) across all datasets, indicating inconsistent performance across data splits.

Among the datasets, CTRPv2 and GDSCv1 exhibit contrasting patterns. CTRPv2 achieves the highest mean $ R^{2} $ computed across models ($ 0.794 $) and notably low prediction variability, suggesting robust data patterns that facilitate accurate and stable predictions. In contrast, GDSCv1 shows lower $ R^{2} $ scores across models ($ 0.694 $), highlighting challenges in capturing strong associations between input features and response data. These findings position CTRPv2 as a relatively stable and predictable dataset for model evaluation, while GDSCv1 presents certain difficulties for obtaining accurate predictions.

The combination of mean $ R^{2} $ and computing standard deviation provides complementary perspectives on model performance. High mean $ R^{2} $ scores reflect a model’s ability to achieve strong predictive accuracy, while low standard deviation indicates stable performance across splits. For example, LGBM combines relatively high predictive performance with good stability, making it a reliable choice for smaller datasets. Conversely, tCNNS underperforms in both predictive accuracy and stability, limiting its utility for within-dataset applications.

These within-dataset results highlight the varying strengths and weaknesses of different models and datasets, providing a foundation for understanding broader cross-dataset trends.

### Cross-dataset performance

The cross-dataset evaluation underscores the inherent challenges in generalizing DRP models to unseen datasets. This can be observed by the more saturated blue shades on the matrix diagonal (representing within-dataset scores) as compared with the off-diagonal (representing cross-dataset scores) in the heatmaps of $ G $ for the different models, shown in Fig. 5. Many entries in these matrices exhibit negative $ R^{2} $ scores (light blue), indicating the inability of models to learn meaningful mappings between input features and treatment response. Despite the substantial differences between within- and cross-dataset scores, certain model-dataset pairs exhibit promising results.

Models such as UNO and GraphDRP achieve relatively better $ R^{2} $ scores in some cross-dataset scenarios compared with others (e.g. UNO: $ G[CTRPv2, CCLE] = 0.631 $, GraphDRP: $ G[CTRPv2, CCLE] = 0.598 $), although performance differences among the top models remain modest. The aggregated cross-dataset metric $ G_{a} $ (Fig. 9) indicates an advantage for UNO and GraphDRP when trained on larger datasets (CTRPv2, GDSCv1, and GDSCv2). LGBM also demonstrates relatively strong generalization on certain dataset pairs, particularly CTRPv2 and GDSCv1, though it shows somewhat reduced performance on GDSCv2. By contrast, tCNNS, HiDRA, and DeepCDR underperform on average compared with others.

**Figure 8:**
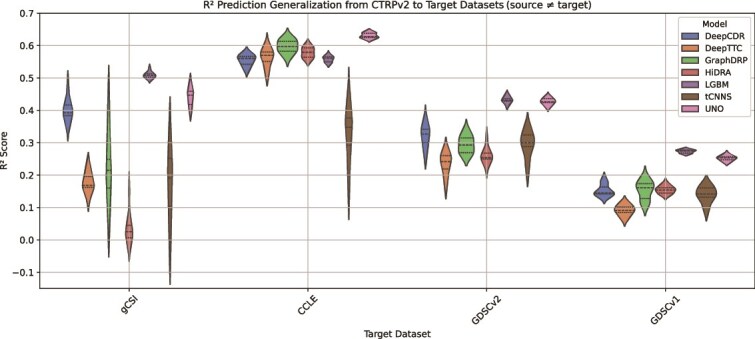
Violin plot showing model generalization performance ($ R^{2} $ scores) when trained on CTRPv2 and evaluated on target datasets (gCSI, CCLE, GDSCv2, and GDSCv1) in the cross-dataset analysis, where each violin plot represents the approximate probability density of the data with width indicating frequency of data points at each $ R^{2} $ value and markers indicating the median and interquartile range (datasets are ordered by total sample size, representing the number of cell–drug response samples).

**Figure 9:**
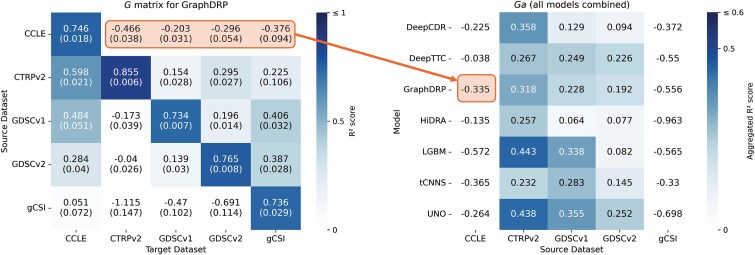
Aggregated cross-dataset performance matrix ($G_{a}$) for seven DRP models (five DL models and one based on LightGBM) and five drug response datasets (Benchmark dataset), with an example average calculation is shown for a $G_{a}[GraphDRP, CCLE] $ entry.

**Figure 10:**
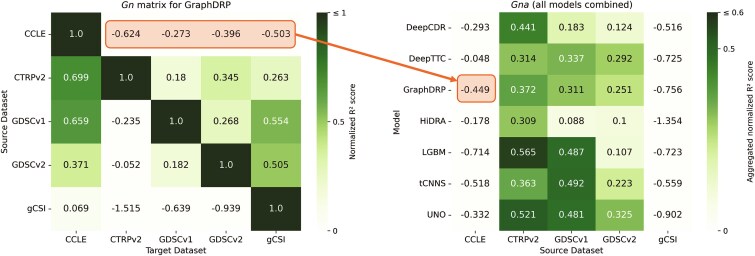
Aggregated Normalized Cross-Dataset Performance matrix (Gna) for seven DRP models (five DL models and one based on LightGBM) and five drug response datasets (Benchmark dataset), with an example average calculation is shown for a $G_{na}[GraphDRP, CCLE] $ entry.

The proposed metrics offer complementary insights into cross-dataset generalization by capturing both absolute performance ($ G $, $ G_{a} $) and relative performance retention ($ G_{n} $, $ G_{na} $) across datasets (see Figs 5 and 10). For example, for GDSCv1, LGBM’s $ G_{a} = 0.3380 $ and $ G_{na} = 0.4868 $ exceed GraphDRP’s $ G_{a} = 0.2284 $ and $ G_{na} = 0.3114 $, with $ G_{na} $ showing a larger gap ($ \Delta G_{na} = 0.4868 - 0.3114 = 0.1754 $ versus $ \Delta G_{a} = 0.3380 - 0.2284 = 0.1096 $), highlighting LGBM’s stronger retention of its within-dataset performance when generalized to other datasets. Similarly, for CTRPv2 $\to $ gCSI, DeepCDR’s $ G = 0.4012 $ and $ G_{n} = 0.4945 $ outperform GraphDRP’s $ G = 0.2248 $ and $ G_{n} = 0.2629 $, with $ G_{n} $ emphasizing the gap ($ \Delta G_{n} = 0.4945 - 0.2629 = 0.2316 $ versus $ \Delta G = 0.4012 - 0.2248 = 0.1764 $), suggesting DeepCDR’s superior cross-dataset performance despite GraphDRP’s higher within-dataset $ R^{2} $ (0.8548 versus 0.8113). However, high $ G_{na} $ values can be misleading when within-dataset performance is low, as seen with tCNNS ($ G_{na}[\mathrm{GDSCv1}] = 0.8587 $, inflated by $ G[\mathrm{GDSCv1}, \mathrm{GDSCv1}] = 0.5745 $). These examples demonstrate how $ G_{n} $ and $ G_{na} $ reveal retention patterns not captured by absolute scores, reinforcing their value as complementary metrics for assessing generalization.

Among the datasets, CTRPv2 emerges as the most effective training source, producing higher generalization scores, highlighting the value of large, high-quality datasets for improving model transferability. The violin plots in Fig. 8 provide a closer look at CTRPv2 as a source dataset, showing its generalization capability to the remaining target datasets. Conversely, gCSI and CCLE consistently result in poor cross-dataset generalization, with mostly negative and close-to-zero $ R^{2} $ scores. These negative $ R^{2} $ values, indicate models perform worse than a naive mean predictor due potential factors such as limited drug diversity (only 16 and 24 unique drugs in gCSI and CCLE, respectively; see Table 1) and model overfitting to source dataset (due to small sample sizes with $<5K$ and $10K$ samples in gCSI and CCLE, respectively; see Table 1), highlighting inherent challenges in using gCSI and CCLE as source datasets.

To contextualize cross-dataset performance, we computed *source-to-target coverage* for drugs (Fig. 6a) and cells (Fig. 6b). For drugs, letting $D_{s}$ and $D_{t}$ denote the sets of unique compounds in source $s$ and target $t$, the drug-set coverage is


\begin{align*} & C_{D}(s,t)=\frac{|D_{s} \cap D_{t}|}{|D_{t}|}. \end{align*}

Analogously for cell lines, with sets $C_{s}$ and $C_{t}$, the cell-line coverage is


\begin{align*} & C_{C}(s,t)=\frac{|C_{s} \cap C_{t}|}{|C_{t}|}. \end{align*}

We can observe that drug-set coverage is strongly associated with cross-dataset performance (off-diagonal values in a $G$ matrix), whereas cell-line coverage shows a positive but generally weaker association. In UNO, e.g. Spearman’s $\rho $ between $C_{D}(s,t)$ and $g(s,t)$ is $0.95$ (Fig. 6c), compared with $0.83$ for $C_{C}(s,t)$ (Fig. 6d). We observe the same pattern across models (drug coverage: $\rho =0.84$–$0.95$; cell coverage: $\rho =0.75$–$0.83$; Supplementary Material S8 provides per-model scatter plots, including the Spearman’s $\rho $ values). The coverage pattern is particularly visible in certain dataset pairs: CTRPv2 to gCSI shows high drug coverage ($C_{D}\!\approx \!0.75$, Fig. 6a) and strong transfer (e.g. UNO: $ G[CTRPv2, gCSI] = 0.44 $, Fig. 5f), whereas gCSI to CTRPv2 exhibits very low drug coverage ($C_{D}\!\approx \!0.02$) and poor transfer (UNO: $ G[gCSI, CTRPv2] = -1.41 $). These observations suggest that drug and cell coverage are strongly associated with generalization.

These results highlight the varying degrees to which different models generalize across datasets, emphasizing the importance of systematic evaluation frameworks. To assess the statistical significance of performance differences, we conducted pairwise Wilcoxon signed-rank tests on $R^{2}$ scores across the seven models for cross-dataset pairs (Supplementary Table S4.1). These tests, alongside boxplots of $R^{2}$ distributions (SupplementaryFig. S4.1), confirm significant differences in model generalization, complementing the proposed metrics ($G$, $G_{a}$, $G_{n}$, and $G_{na}$) for a nuanced perspective on transferability across datasets.

## Discussion

In this work, we present a benchmarking framework for assessing the prediction generalization capabilities of DRP models. The framework incorporates five drug response datasets (Section Benchmark dataset), six DRP models implemented within a unified code structure (Section Models), software tools enabling scalable execution of the evaluation workflow (Section Cross-dataset workflow), and four metrics designed to assess the generalization capabilities of models (Section Performance metrics). As part of the IMPROVE project [11], this study underscores both the importance and challenges of rigorous benchmarking, with a particular emphasis on evaluating cross-dataset generalization, an essential but underexplored aspect of DRP model assessment.

Our key findings reveal substantial disparities between within- and cross-dataset performance. All models exhibit a significant drop in prediction accuracy when applied to external datasets, highlighting the persistent challenge of out-of-distribution (OOD) generalization. However, certain models, such as UNO, GraphDRP, and LGBM, demonstrate moderately better performance within individual datasets and retain a portion of their predictive capability across datasets, although differences among these top models remain subtle. From the dataset perspective, CTRPv2 emerges as the most effective source dataset, consistently producing higher generalization scores across multiple target datasets. This is likely due to its larger sample size and greater diversity in both unique cell lines and drug compounds, factors that contribute to better generalization. Moreover, the proposed evaluation metrics (see Section Performance metrics) provide complementary insights into both absolute and relative generalization capabilities. These metrics prove instrumental in identifying robust models while also highlighting those that exhibit the most pronounced decline in cross-dataset generalization relative to their within-dataset performance (see Fig. 10).

Assessing generalization across drug response datasets is not a novel concept in DRP research. Many recent studies acknowledge the limitations of within-dataset evaluation and attempt to validate their models using a single external dataset. However, such validation strategy may not fully capture the complexities of model transferability across contexts. Furthermore, our prior work has already explored cross-dataset generalization through similar $ d $-by-$ d $ evaluations [9, 36]. Our current study builds upon these efforts by introducing a structured benchmarking framework designed to enable fair and scalable comparisons of models. By ensuring consistent data preprocessing, standardized evaluation metrics, and rigorous experimental design, this framework provides a more reliable means of assessing model robustness in cross-dataset scenarios. We emphasize that the goal of this work is not to demonstrate the superiority of a particular model but rather to present a framework that highlights the challenges associated with cross-dataset analysis. A structured evaluation methodology is critical for accurately assessing the reliability and transferability of AI-based DRP models into real-world biomedical applications.

The observed cross-dataset performance degradation may stem from OOD generalization challenges as well as dataset-specific experimental differences. For instance, differences in viability assays, such as GDSCv1’s use of Syto60 versus CellTiter-Glo in CCLE, CTRPv2, gCSI, and GDSCv2, can substantially affect response values and hinder model transferability [9]. Consistent with prior scaling experiments highlighting the role of drug diversity in transferability [9], our source-to-target coverage analysis shows that compound coverage is strongly associated with cross-dataset performance (off-diagonal values in a $G$ matrix), whereas cell-line coverage is positive but generally weaker. We note that coverage is not the only factor: experimental differences (e.g. assay protocols and processing), overall sample size, and data quality are also likely contributors. Addressing these challenges will likely require tailored strategies, such as increasing compound overlap with the intended target, employing transfer learning techniques, harmonizing assays where feasible, or constructing drug-specific models that are likely to be less sensitive to limited compound diversity. The modularity and flexibility of our benchmarking framework facilitate identifying such generalization challenges and evaluating mitigation strategies.

Architectural choices likely contribute to performance differences, particularly when considered alongside dataset-specific factors like drug diversity and sample size (Table 1). On CTRPv2, a large dataset with high compound diversity ($\sim 500$ drugs, $\sim 286$ K responses), models such as LGBM, UNO, DeepCDR, and GraphDRP demonstrate robust performance, with strong within-dataset and good cross-dataset results. LGBM, a tree-based ensemble method, may generalize well without extensive tuning due to its inherent robustness, as supported by SHAP analysis showing consistent reliance on key drug descriptors and gene expression features across datasets (Supplementary Material S5). UNO, which uses MLP-based subnetworks for gene expression and drug inputs, may benefit from residual connections and dropout layers in the prediction network, aiding in learning complex patterns while mitigating overfitting. DeepCDR and GraphDRP, incorporating GNNs for drug representation, may better capture drug molecular topologies, particularly with structurally diverse drug sets such as CTRPv2. DeepTTC, with transformer-based encoders for drug FPs, excels within-dataset but shows modest cross-dataset results, possibly reflecting high model complexity relative to dataset scale. HiDRA’s attention-based design may support interpretability, though it performs moderately within-dataset with limited cross-dataset generalization [10, 33]. A concise, model summary of empirical capabilities and plausible limitations appears in Supplementary Material S9.

This study has several important limitations. First, the datasets were obtained from different studies, each potentially contributing unique biases stemming from varying experimental conditions and assay protocols. Differences in data generation and collection can impact model performance and adversely impact generalizability. Second, while the standardized code for training and inference follows a similar pattern across models, the preprocessing stage remains highly model-dependent. Establishing more structured guidelines for preprocessing would enhance standardization and improve the reliability of benchmarking. Third, we employed random data partitioning to obtain the data splits, which can lead to inflated performance due to potential data leakage across cell line and drugs. Future work should explore more stringent splitting strategies, such as drug-blind and cell-blind splits, to better simulate real-world scenarios [3]. Finally, the reported results were obtained using limited hyperparameter optimization (HPO). While our primary objective is to examine broad trends and generalization patterns rather than optimizing predictive accuracy, a more exhaustive HPO search could refine model performance. Consequently, while the results highlight performance trends, definitive statements about the superiority of specific models should be interpreted cautiously.

Future work could build upon this benchmarking framework by exploring learning techniques such as transfer learning [8, 14, 16], including domain adaptation [7, 17, 38] to mitigate the impact of OOD predictions, thereby improving cross-dataset performance and narrowing the gap between within- and cross-dataset generalization. While the current study exclusively uses omics feature data from DepMap to control for variation in cancer features, real-world scenarios may involve predicting on target datasets that provide their own feature data (e.g. training on cell lines and predicting on PDX or organoids). In such cases, correcting for potential batch effects becomes essential [39–41]. Additionally, alternative molecular data sources, such as COSMIC [42], could be explored to assess how the choice of omics repository impacts the observed performance of DRP models. Given the modular design of our framework, these extensions can be systematically evaluated in future studies. For multimodal models like DeepCDR and UNO, the framework’s flexible parameter interface could facilitate ablation studies to assess the contribution of individual input modalities, provided the model architectures allow activation or deactivation of specific feature types. Another promising direction involves extending the evaluation scheme beyond single-valued generalization metrics to alternative assessments, such as studying how model performance scales with increasing data availability [20, 43], comparing model interpretability for clinical decision-making [10], or integrating evaluation methodologies relevant to drug discovery applications [44, 45]. Additionally, data-centric approaches have proven effective in enhancing prediction accuracy in traditional ML benchmarks, making them particularly valuable in biomedical applications where data scarcity is a major challenge. A key future direction is to systematically construct training and evaluation sets to align more closely with the data distribution of target datasets, ensuring that models are better suitable for real-world deployment in preclinical and clinical settings.

## Conclusion

This study highlights the pressing need for rigorous benchmarking in DRP model development. Rather than identifying a single superior model, our framework enables fair and meaningful model comparisons, emphasizing the necessity of structured evaluation methodologies that reflect real-world complexities in predictive oncology. Scientists across various domains continue to adapt advanced AI techniques for diverse applications, yet without rigorous benchmarking practices, it remains challenging to assess the true potential and limitations of these approaches [46–49]. Robust benchmarking frameworks are indispensable for assessing the true potential of prediction models, cutting through hype, fostering trust, and bridging the crucial gap between AI-based DRP models and their adoption in preclinical and clinical applications.

**Key Points**

- We introduce a scalable benchmarking framework to evaluate the generalization patterns of drug response prediction (DRP) models across datasets, aiming to identify potential challenges related to both models and datasets.
- The framework is demonstrated through standardized training and evaluation of seven curated DRP models across five public drug screening datasets.
- We propose novel metrics that quantify both absolute predictive performance and relative performance degradation when models are applied to unseen datasets.
- Our results show significant performance degradation in cross-dataset scenarios, emphasizing the critical need for rigorous model evaluation practices in DRP.
- All datasets, models, and workflows are publicly released to facilitate community-wide benchmarking efforts.

## Supplementary Material

Supplementary_Materials_bbaf667

## Data Availability

The reproducibility package for this study is available at github.com/adpartin/cross-dataset-drp-paper, with the exact version used for all results archived under the tagged release v1.2-bib-paper-repro and preserved on Zenodo at 10.5281/zenodo.15258742. The benchmark dataset, including drug response data, omics features, drug representations, and predefined data splits, is archived at 10.5281/zenodo.15258883. The model implementations and cross-dataset analysis workflow are versioned for this study using the tag v0.1.0 across the corresponding repositories, as described in the reproducibility package linked above.
