# Supplementary material for: Benchmarking community drug response prediction models: datasets, models, tools, and metrics for cross-dataset generalization analysis
Source: Brief Bioinform. 2026 Jan 12;27(1):bbaf667. doi: 10.1093/bib/bbaf667 (PMC12794626; doi:10.1093/bib/bbaf667)
Supplement: Supplementary_Materials_bbaf667 [file supplementary_materials_bbaf667.pdf]

Supplementary Material S1

Table S1 compares studies that evaluate model performance, including cross-dataset prediction, interpretability (Li et al., 2023), and IC50 vs. AAC prediction (Sharifi-Noghabi et al., 2021). These studies conduct rigorous analyses with multiple models, yielding significant insights for drug response prediction (DRP). While all studies provide reproducibility resources, Sharifi-Noghabi et al. and Xia et al. lack guidelines for adding models or datasets, and Li's et al. modularity is limited to model integration. Our study introduces a modular framework (IMPROVE) enabling extensions with new datasets, models, and evaluation schemes.

Abbreviations: PCC (Pearson correlation coefficient), RMSE (root mean squared error), RF (random forest), ENet (elastic net), RidgeReg (ridge regression), DL (deep learning), ML (machine learning), NN (neural network), FPs (fingerprints), GEx (gene expression), CNV (copy-number variation), Mut (mutation), LPO (leave-pairs-out), LCO (leave-cell-lines-out), and LDO (leave-drugs-out), PPI (protein-protein interactions).

Table S1. Available model comparison studies.

Study	Assessment Goal	Data Sources	Models	Evaluation Approaches	Tool Availability
Sharifi-Noghabi et al. (2021)	Evaluates prediction performance of ML models in within- and cross-study settings, comparing IC50 vs. AAC.	GEx data from CCLE, GDSC via PharmacoDB; no drug features; response data (IC_{50}, AAC) from CTRPv2, gCSI, GDSCv1, GDSCv2.	Four models (ML: ENet, RF, RidgeReg; DL: NN), trained per drug on omics data only (no drug features).	Within-study and cross-study evaluation settings with standard metrics such as PCC and RMSE.	Resources for reproducibility include: GitHub repo, Code Ocean capsule, Zenodo; limited guidelines on adding new models.
Xia et al. (2022)	Compares cross-dataset generalization across 5 datasets and with ML and DL models.	GEx profiles from CCLE; molecular descriptors and FPs as drug features; response data from CCLE, CTRP, gCSI, GDSC, NCI60.	Three models (ML: RF, LightGBM; DL: UnoMT); predicting dose-dependent responses.	Cross-dataset heatmaps with R^2 and MAE; learning curve for analyzing the effect of drug/cell-line diversity on cross-dataset performance.	Github repo with UnoMT; datasets in FTP; limited documentation on reproducibility.
Li et al. (2023)	Evaluates prediction performance and interpretability across DL models and multiomics datasets	GEx, CNV, Mut from GDSC; drug targets (STITCH); PPI (STRING); Gene-pathway (KEGG); IC50 from GDSC.	Six models (ML: RF; DL: MLP, PathDNN, CDS, HiDRA, PathDSP; drug target and FPs used as drug information.	LPO, LCO, LDO evaluation strategies; interpretability via feature attribution.	Resources for reproducibility include: datasets in Zenodo, code in Github repo; modular scripts for adding new models, but limited integration of new datasets or evaluation schemes
Our Study	Systematic evaluation of cross-dataset prediction performance of DL and ML models	Multiomics data were obtained from CCLE (total of 10 types as described below); drug features include 3 types (as described below); response data from CCLE, CTRPv2, gCSI, GDSCv1, GDSCv2.	LightGBM and six modern DL-based DRP models (DeepCDR, DeepTTC, GraphDRP, HiDRA, tCNNS, UNO)	Cross-dataset heatmaps with R^2 and standard deviation across data splits.	Resources for reproducibility include: datasets in Zenodo, and Github repos includes models and code; comprehensive IMPROVE documentation enables adding datasets, models, evaluation schemes

The multiomics data, drug representations, and drug responses mentioned in the table in Our Study represent the benchmark data, and include the following files (https://zenodo.org/records/15258883).
Multiomics representations:

1. cancer_DNA_methylation.tsv: average DNA methylation values in transcription start sites.
2. cancer_RPPA.tsv: protein expressions measured using RPPA.
3. cancer_copy_number.tsv: continuous gene copy numbers.

4. cancer_discretized_copy_number.tsv: discretized gene copy numbers. -2, -1, 0, 1, 2 indicate deep deletion, heterozygous deletion, neutral, copy number gain, copy number amplification, respectively.
5. cancer_gene_expression.tsv: log2 transformed TPM (transcripts per million reads mapped) gene expression values.
6. cancer_miRNA_expression.tsv: miRNA expression values.
7. cancer_mutation.parquet: binary matrix indicating mutation occurrences. Columns are cell lines. Rows are mutations.
8. cancer_mutation_count.tsv: gene-level mutation counts in cell lines.
9. cancer_mutation_long_format.tsv: each row indicates the occurrence of a mutation in a cell line.
10. drug_SMILES.tsv: SMILES strings of drugs.

Drug representations:
1. drug_ecfp4_nbits512.tsv: ecfp4 binary fingerprints of drugs.
2. drug_info.tsv: meta information of drugs.
3. drug_mordred.tsv: Mordred descriptors of drugs.

References

1. H. Sharifi-Noghabi et al. “Drug sensitivity prediction from cell line-based pharmacogenomics data: Guidelines for developing machine learning models.” Briefings in Bioinformatics, 22(6), November 2021, https://academic.oup.com/bib/article/22/6/bbab294/6348324
2. F. Xia et al. “A cross-study analysis of drug response prediction in cancer cell lines.” Briefings in Bioinformatics, 23(1), 2022, https://academic.oup.com/bib/article/23/1/bbab356/6370300
3. Y. Li et al. “Interpretable deep learning architectures for improving drug response prediction performance: myth or reality?” Bioinformatics, 39(6), 2023, https://academic.oup.com/bioinformatics/article/39/6/btad390/7199590

Supplementary Material S2

This section provides a concrete example of Supplementary Data (*Supp. Data*) used by the HiDRA model (see Fig 3. in the main text).

HiDRA uses supplementary gene set data from KEGG pathways, sourced from the Molecular Signatures Database (MSigDB), to structure gene expression features into 186 pathway-level representations. The path to this data is specified by setting the parameter `input_supp_data_dir` in the parameter configuration file. For HiDRA, this parameter is set to `author_data`, as shown in the parameter file:
https://github.com/JDACS4C-IMPROVE/HiDRA/blob/develop/hidra_params.txt

The `author_data` directory contains the file `geneset.gmt`. This file contains gene sets (i.e., list of gene symbols) with 186 KEGG pathways and their member genes (4,592 unique genes). This file enables HiDRA to aggregate gene expression data into pathway-level features for its hierarchical attention network. The preprocessing script integrates `geneset.gmt` with benchmark gene expression data to create model-ready inputs. The KEGG pathway data is publicly available via MSigDB.

A partial directory structure is shown below, highlighting the `author_data` directory for HiDRA's *Supp. Data* and a subset of the benchmark data (`csa_data`):

```
HiDRA/
├── author_data/
│   └── geneset.gmt
└── csa_data/
    ├── x_data/
    │   ├── cancer_gene_expression.tsv
    │   ├── cancer_mutation_count.tsv
    │   ├── ...
    │   ├── drug__mordred.tsv
    │   └── drug_SMILES.tsv
    ├── y_data/
    │   └── response.tsv
    └── splits/
        ├── CCLE_split_0_train.txt
        ├── CCLE_split_0_val.txt
        ├── CCLE_split_0_test.txt
        ├── ...
        ├── CCLE_split_9_train.txt
        ├── CCLE_split_9_val.txt
        ├── CCLE_split_9_test.txt
        ├── ...
        ├── GDSCv2_split_0_train.txt
        ├── GDSCv2_split_0_val.txt
        ├── GDSCv2_split_0_test.txt
        ├── ...
        ├── GDSCv2_split_9_train.txt
        ├── GDSCv2_split_9_val.txt
        └── GDSCv2_split_9_test.txt
```

Supplementary Material S3

This section complements Figures 5 and 6 in the main text with additional tables and figures. Fig. 5 presents the cross-dataset performance matrix (G), showing mean R^2 scores for within- and cross-dataset cases. Fig. 6 visualizes within-dataset R^2 distributions via violin plots. Tables S3.1 and S3.2 report within-dataset mean R^2 and standard deviation, respectively, corresponding to Fig. 5 diagonal entries. Fig. S3.1 provides a bubble heatmap of within-dataset performance, with color encoding mean R^2, bubble size representing inverse variance ($1/\sigma^2$), and text annotations for exact values, offering an alternative visualization to Tables S3.1/S3.2 and Fig. 6.

Table S3.1. Within-Dataset Analysis (mean R^2). Each value represents the mean R^2 computed across 10 data splits for a model-dataset pair, corresponding to the diagonal entries in Fig. 5 G matrices. Rows reflect datasets ordered by increasing sample size (gCSI to CTRPv2). In addition, "Mean across datasets" and "Mean across models" summarize the average performance across datasets and models, respectively.

Model	gCSI	CCLE	GDSCv2	GDSCv1	CTRPv2	Mean across models
**DeepCDR**	0.72	0.766	0.76	0.704	0.811	0.752
**GraphDRP**	0.736	0.746	0.765	0.733	0.855	0.767
**HiDRA**	0.711	0.756	0.768	0.722	0.832	0.758
**LGBM**	0.782	0.801	0.764	0.695	0.784	0.765
**tCNNS**	0.591	0.705	0.648	0.575	0.639	0.632
**UNO**	0.774	0.796	0.775	0.738	0.841	0.785
**Mean across datasets**	0.719	0.762	0.747	0.695	0.794	

Table S3.2. Within-Dataset Analysis (Standard Deviation of R^2). Each value represents the standard deviation of R^2 computed across 10 data splits for a model-dataset pair, corresponding to the values in parentheses of the diagonal entries in Fig. 5 G matrices. Rows reflect datasets ordered by increasing sample size (gCSI to CTRPv2). In addition, "Mean across datasets" and "Mean across models" summarize the average variability across datasets and models, respectively.

Model	gCSI	CCLE	GDSCv2	GDSCv1	CTRPv2	Mean across models
**DeepCDR**	0.02	0.023	0.007	0.008	0.005	0.0126
**GraphDRP**	0.029	0.018	0.008	0.007	0.006	0.0136
**HiDRA**	0.027	0.02	0.011	0.007	0.005	0.0140
**LGBM**	0.02	0.011	0.008	0.006	0.003	0.0096
**tCNNS**	0.061	0.049	0.052	0.049	0.063	0.0548
**UNO**	0.025	0.012	0.007	0.007	0.006	0.0114
**Mean across datasets**	0.03	0.02	0.0155	0.014	0.015	

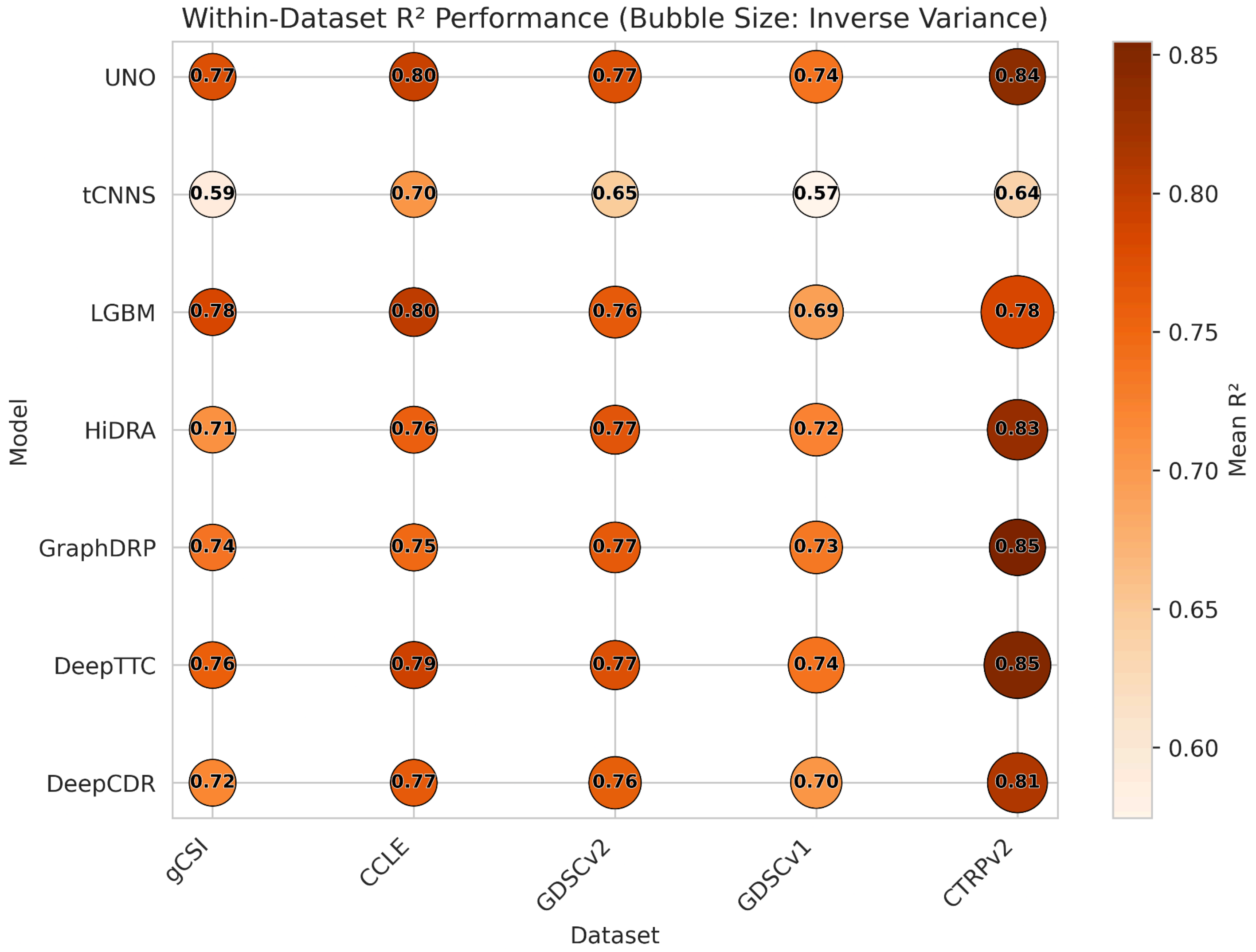

<u>Fig. S3.1. Bubble Heatmap of Within-Dataset Performance.</u> This visualization represents mean R^2 scores and inverse variance (larger bubble size for lower variance, i.e., more stable) for 7 models across 5 datasets, ordered by increasing sample size (gCSI to CTRPv2). Text annotations provide exact mean R^2 values, complementing Tables S3.1 and S3.2 and violin plots in Fig. 6.

Supplementary Material S4

Table S4.1. Wilcoxon Signed-Rank Tests. Pairwise Wilcoxon signed-rank tests compare R^2 performance across seven models (DeepCDR, DeepTTC, GraphDRP, HiDRA, LGBM, tCNNS, UNO) for cross-dataset pairs (source ≠ target). We selected comparisons with $p < 0.00238$ (Bonferroni-corrected for 21 model comparisons; C(7,2)=21) and where at least one model's median $R^2 > 0.1$ across 10 splits, choosing up to three comparisons per source-target pair with the largest absolute mean R^2 differences, yielding 36 comparisons. Columns show source and target datasets, models compared, median R^2 values (across 10 data splits), and mean R^2 difference (models1 - model2, between the corresponding splits). Full results are available at https://zenodo.org/records/15258742.

Source	Target	Model 1	Model 2	Median Model 1	Median Model 2	Mean R2 difference
CCLE	gCSI	GraphDRP	UNO	-0.3830	0.2001	-0.5670
CCLE	gCSI	tCNNS	UNO	-0.1137	0.2001	-0.5067
CCLE	gCSI	LGBM	UNO	-0.1191	0.2001	-0.3086
CTRPv2	CCLE	tCNNS	UNO	0.3477	0.6279	-0.3068
CTRPv2	CCLE	GraphDRP	tCNNS	0.5974	0.3477	0.2735
CTRPv2	CCLE	HiDRA	tCNNS	0.5793	0.3477	0.2544
CTRPv2	GDSCv1	DeepTTC	LGBM	0.0912	0.2747	-0.1812
CTRPv2	GDSCv1	DeepTTC	UNO	0.0912	0.2533	-0.1600
CTRPv2	GDSCv1	LGBM	tCNNS	0.2747	0.1416	0.1338
CTRPv2	GDSCv2	DeepTTC	LGBM	0.2419	0.4316	-0.1968
CTRPv2	GDSCv2	DeepTTC	UNO	0.2419	0.4265	-0.1919
CTRPv2	GDSCv2	HiDRA	LGBM	0.2540	0.4316	-0.1717
CTRPv2	gCSI	HiDRA	LGBM	0.0256	0.5080	-0.4734
CTRPv2	gCSI	HiDRA	UNO	0.0256	0.4468	-0.4064
CTRPv2	gCSI	DeepCDR	HiDRA	0.3926	0.0256	0.3658
GDSCv1	CCLE	HiDRA	UNO	0.2922	0.5744	-0.2822
GDSCv1	CCLE	DeepCDR	HiDRA	0.5230	0.2922	0.2320
GDSCv1	CCLE	tCNNS	UNO	0.3462	0.5744	-0.2280
GDSCv1	GDSCv2	HiDRA	LGBM	0.0753	0.3499	-0.2762
GDSCv1	GDSCv2	HiDRA	UNO	0.0753	0.3244	-0.2531
GDSCv1	GDSCv2	DeepCDR	LGBM	0.1008	0.3499	-0.2409
GDSCv1	gCSI	HiDRA	LGBM	0.1952	0.5519	-0.3851
GDSCv1	gCSI	DeepCDR	LGBM	0.2164	0.5519	-0.3275
GDSCv1	gCSI	HiDRA	tCNNS	0.1952	0.4975	-0.3255

GDSCv2	CCLE	GraphDRP	LGBM	0.2762	-0.0175	0.3021
GDSCv2	CCLE	DeepCDR	GraphDRP	-0.0180	0.2762	-0.2852
GDSCv2	CCLE	GraphDRP	HiDRA	0.2762	0.0025	0.2838
GDSCv2	CTRPv2	DeepTTC	LGBM	0.1341	-0.3704	0.5011
GDSCv2	CTRPv2	LGBM	tCNNS	-0.3704	0.1333	-0.4773
GDSCv2	CTRPv2	DeepCDR	DeepTTC	-0.1568	0.1341	-0.3175
GDSCv2	GDSCv1	HiDRA	UNO	0.1102	0.1881	-0.0760
GDSCv2	GDSCv1	DeepTTC	UNO	0.1268	0.1881	-0.0643
GDSCv2	GDSCv1	tCNNS	UNO	0.1385	0.1881	-0.0539
GDSCv2	gCSI	tCNNS	UNO	0.2908	0.5551	-0.2621
GDSCv2	gCSI	LGBM	tCNNS	0.5550	0.2908	0.2613
GDSCv2	gCSI	HiDRA	UNO	0.3095	0.5551	-0.2463

<u>Figure S4.1. Boxplots of R^2 Scores.</u> Boxplots show R^2 distributions across 10 splits for 12 cross-dataset pairs (source ≠ target) where at least one model has mean $R^2 \geq 0.1$, excluding source-target pairs where all models had mean $R^2 < 0.1$, indicating poor performance (no variance explained). Distributions show R^2 values across 10 splits with means (black dots) and medians (lines). See Table S4.1 for significant comparisons.

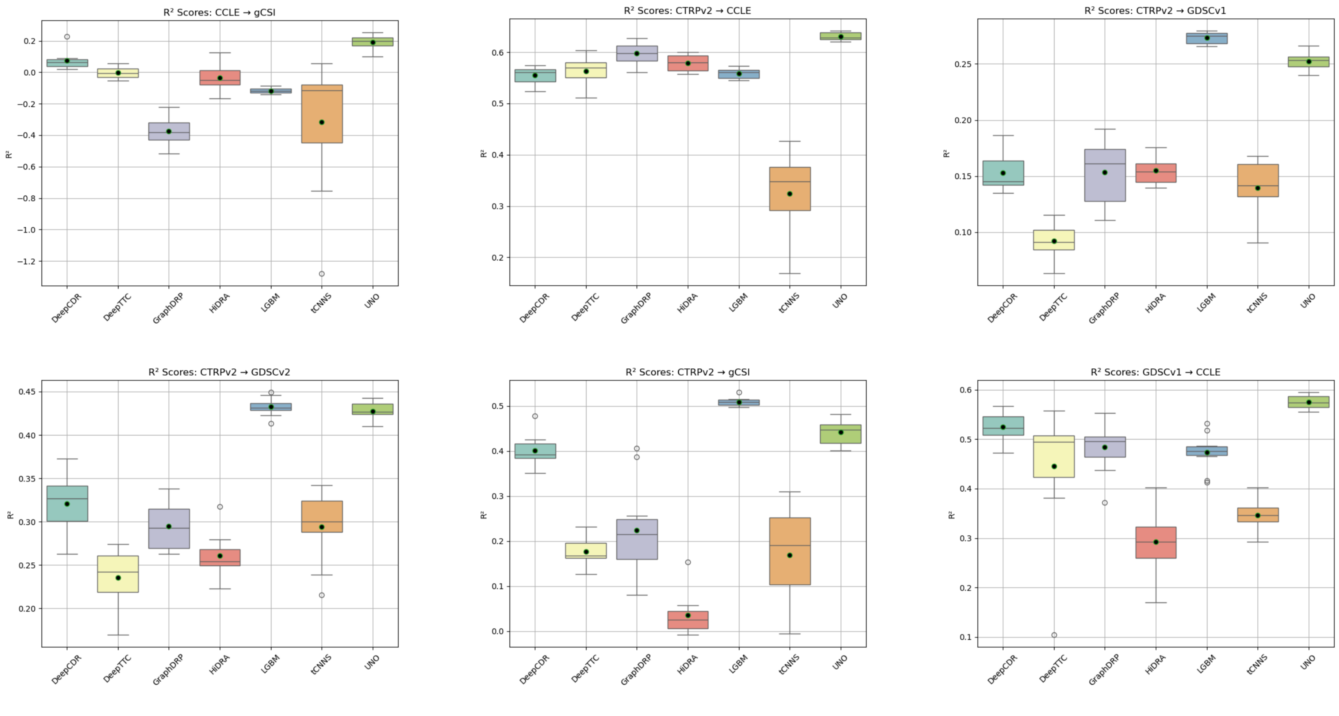

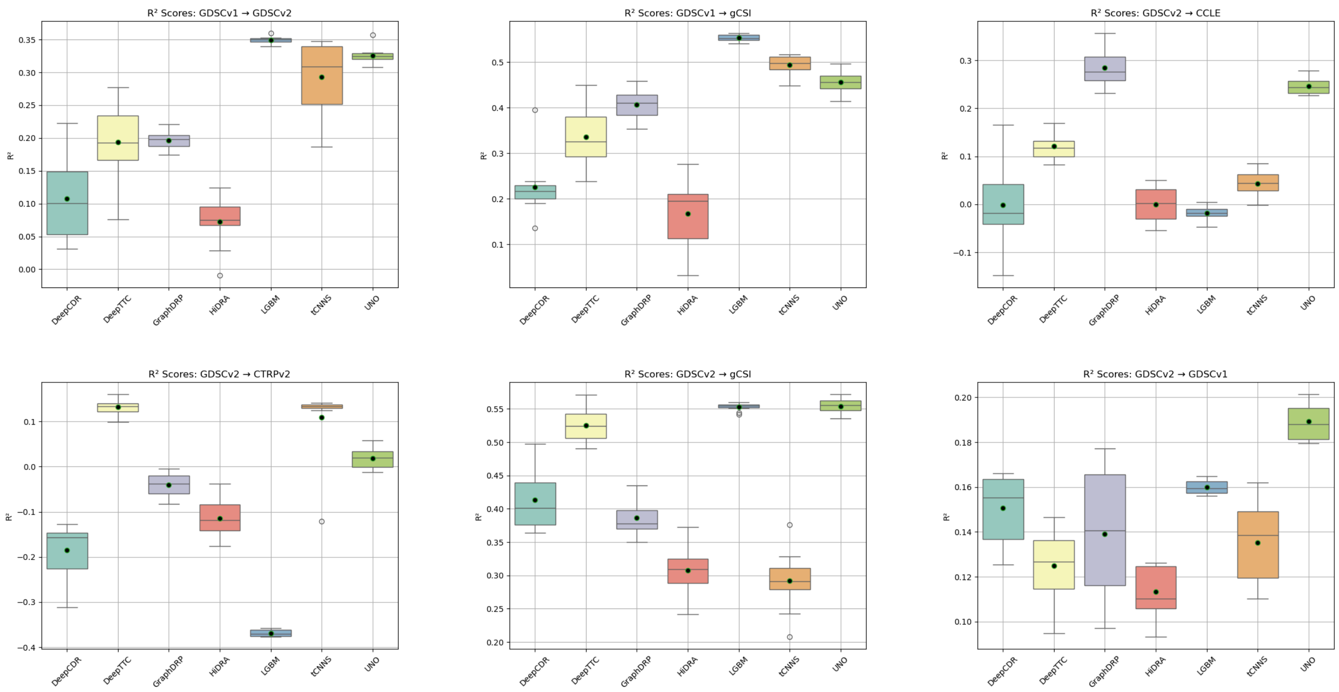

R² Scores: GDSCv1 → GDSCv2
R² Scores: GDSCv1 → gCSI
R² Scores: GDSCv2 → CCLE
R² Scores: GDSCv2 → CTRPv2
R² Scores: GDSCv2 → gCSI
R² Scores: GDSCv2 → GDSCv1
R²
DeepCDR
DeepTTC
GraphDRP
HiDRA
LGBM
tCNNS
UNO

Supplementary Material S5

Figure S5. SHAP plots. SHAP analysis of LGBM feature attributions across three dataset-pair scenarios, showing the top 25 features in each case. Summary plots (left) illustrate feature-level contributions and value distributions; bar plots (right) rank features by mean absolute SHAP value. In well-generalizing settings (CTRPv2→CTRPv2 and CTRPv2→gCSI), top-ranked features show substantial overlap and comprise both drug Mordred descriptors and gene expression (GE) features. In contrast, the poorly generalizing case (CCLE→gCSI) exhibits minimal feature overlap, suggesting inconsistent reliance on predictive features across domains.

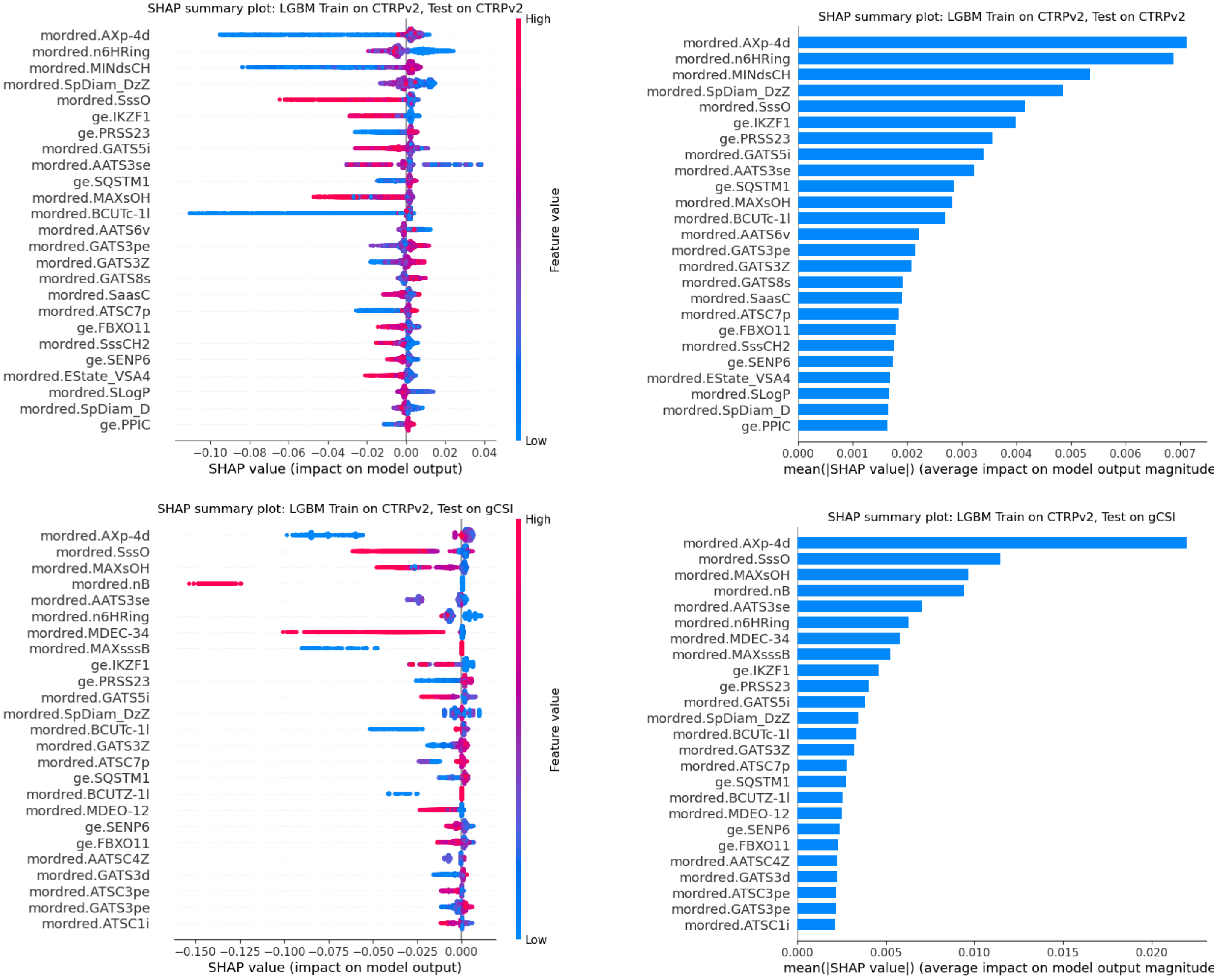

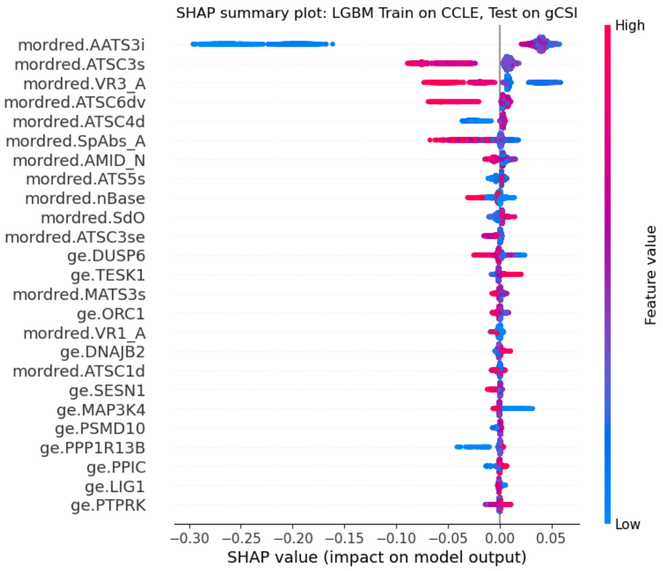
SHAP summary plot: LGBM Train on CCLE, Test on gCSI
mordred.AATS3i
mordred.ATSC3s
mordred.VR3_A
mordred.ATSC6dv
mordred.ATSC4d
mordred.SpAbs_A
mordred.AMID_N
mordred.ATS5s
mordred.nBase
mordred.SdO
mordred.ATSC3se
ge.DUSP6
ge.TESK1
mordred.MATS3s
ge.ORC1
mordred.VR1_A
ge.DNAJB2
mordred.ATSC1d
ge.SESN1
ge.MAP3K4
ge.PSMD10
ge.PPP1R13B
ge.PPIC
ge.LIG1
ge.PTPRK
−0.30 −0.25 −0.20 −0.15 −0.10 −0.05 0.00 0.05
SHAP value (impact on model output)
High
Feature value
Low

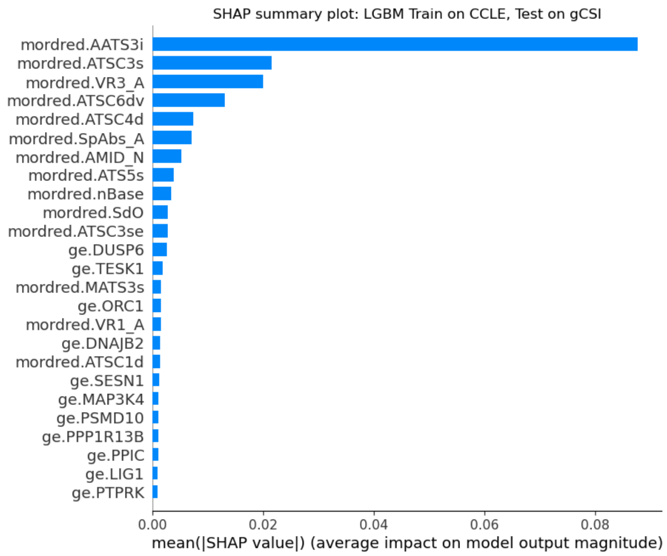
SHAP summary plot: LGBM Train on CCLE, Test on gCSI
mordred.AATS3i
mordred.ATSC3s
mordred.VR3_A
mordred.ATSC6dv
mordred.ATSC4d
mordred.SpAbs_A
mordred.AMID_N
mordred.ATS5s
mordred.nBase
mordred.SdO
mordred.ATSC3se
ge.DUSP6
ge.TESK1
mordred.MATS3s
ge.ORC1
mordred.VR1_A
ge.DNAJB2
mordred.ATSC1d
ge.SESN1
ge.MAP3K4
ge.PSMD10
ge.PPP1R13B
ge.PPIC
ge.LIG1
ge.PTPRK
0.00 0.02 0.04 0.06 0.08
mean(|SHAP value|) (average impact on model output magnitude)

Supplementary Material S6

Figure S6. G and Gn heatmaps for tCNNs. These correspond to the performance matrices shown in Fig. 5 for other models. tCNNs were moved to the supplementary material to preserve space and improve clarity.

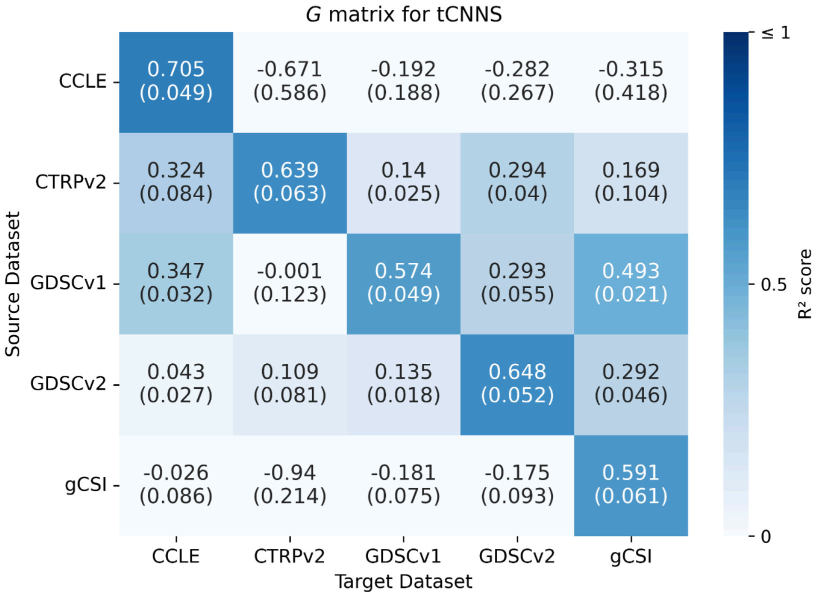

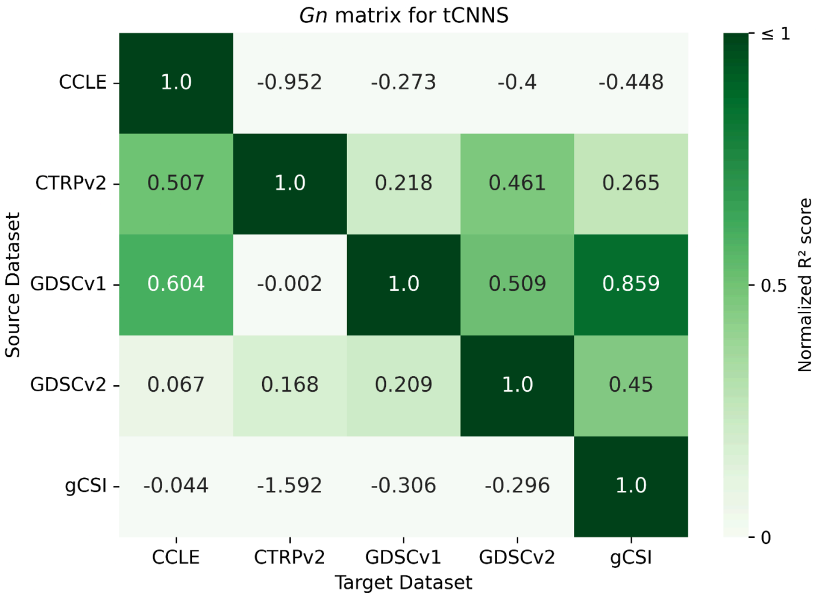

Supplementary Material S7

This guide describes main components and I/O of the preprocess, train, and infer stages, the file-naming conventions, and LLM-based utility tool that assists with model standardization. A worked example with LGBM model is provided. Once a model is standardized in accordance with improve compliance standards, any workflow within the improvelib library (e.g., CSA) can be run with the model. For in-depth documentation, see the library docs: https://jdacs4c-improve.github.io/docs/content/curating.html.

Figure S7.1. Required files and naming convention.
https://jdacs4c-improve.github.io/docs/content/curating_compliance.html#required-files-and-their-naming-conventions

Files required for compliance:
- `<model>_preprocess_improve.py`
- `<model>_train_improve.py`
- `<model>_infer_improve.py`
- `<model>_params.ini`
- `model_params_def.py`
- `<model>_environment.yml`

Files required for compliance (LGBM):
- `lgbm_preprocess_improve.py`
- `lgbm_train_improve.py`
- `lgbm_infer_improve.py`
- `lgbm_params.ini`
- `model_params_def.py`
- `lgbm_environment.yml`

Figure S7.2. Preprocessing. Essential components of the preprocessing script, including imports, raw benchmark data loading and processing, and saving the model-ready data. Additionally, the inputs and outputs to and from this process are shown. The script takes raw benchmark data, including feature data (x data), response data (y data), and pre-computed data splits, and generate model-ready data files for the prediction model. The naming convention for the preprocessing script is `<model>_preprocess_improve.py.` Refer to documentation for detailed description:
https://github.com/JDACS4C-IMPROVE/LGBM/blob/develop/lgbm_preprocess_improve.py

Benchmark Data

X Data:
- `response.tsv`

Y Data:
- `cancer_gene_expression.tsv`
- `drug_mordred.tsv`

Splits:
- `CCLE_split_0_train.txt`
- `CCLE_split_0_val.txt`
- `CCLE_split_0_test.txt`

Preprocessing (code snippets)

Essential Imports:
```
# Core improvelib imports
import improvelib.utils as frm
# Application-specific (DRP) imports
from improvelib.applications.drug_response_prediction.config import (
DRPPreprocessConfig)
import improvelib.applications.drug_response_prediction.drp_utils as drp
# Model-specifc imports
from model_params_def import preprocess_params
```

Data Loading:
```
omics = frm.get_x_data(file = params['cell_transcriptomic_file'], …)
drugs = frm.get_x_data(file = params['drug_mordred_file'])
response_train = frm.get_y_data(split_file=params["train_split_file"])
```

Data Preparation:
```
# model-specific steps
```

Save Data:
```
frm.save_stage_ydf(ydf, stage, params["output_dir"])
```

Processed Data

Processed X Data:
- `train_data.parquet`
- `val_data.parquet`
- `test_data.parquet`

Processed Y Data:
- `train_y_data.csv`
- `val_y_data.csv`
- `test_y_data.csv`

Other:
- `param_log_file.txt`
- `runtime_preprocess.json`

Figure S7.3. Training. Essential components of the train script, including imports, model-ready data loading, training and saving of the model, and saving model predictions and performance scores. Additionally, the inputs and outputs to and from this process are shown. The script takes train and val model-ready data, trains the model (with early stopping on val data), and saves the trained model and performance scores computed on val data. The naming convention for the training script is `<model>_train_improve.py.` Refer to documentation for detailed description:

https://github.com/JDACS4C-IMPROVE/LGBM/blob/develop/lgbm_train_improve.py

Model-Ready Data

Processed X Data:
- `train_data.parquet`
- `val_data.parquet`
- `test_data.parquet`

Processed Y Data:
- `train_y_data.csv`
- `val_y_data.csv`
- `test_y_data.csv`

Training (code snippets)

Essential Imports:
```
# Core improvelib imports
import improvelib.utils as frm
# Application-specific (DRP) imports
from improvelib.applications.drug_response_prediction.config import (
DRPTrainConfig)
# Model-specifc imports
from model_params_def import train_params
import lightgbm as lgb
```

Data Loading:
```
# model-specific
train_data = pd.read_parquet(Path(params["input_dir"]) / train_data_fname)
val_data = pd.read_parquet(Path(params["input_dir"]) / val_data_fname)
```

Train and Save Model:
```
# model-specific
model.fit(xtr, ytr, eval_set=[(xvl, yvl)])
model.save_model(str(modelpath))
```

Save Predictions and Scores:
- `frm.store_predictions_df(…)`
- `val_scores = frm.compute_performance_scores(…)`

Model

Trained Model:
- `model.json`

Val Predictions:
- `val_y_data_predicted.csv`

Val Scores:
- `val_scores.csv`

Other:
- `param_log_file.txt`
- `runtime_train.json`

<u>Figure S7.4. Inference.</u> Essential components of the inference script, including imports, model-ready data loading, and running inference on the test data. Additionally, the inputs and outputs to and from this process are shown. The script takes test model-ready data and the trained model, runs the model in inference mode, and saves the predictions and performance scores computed on test data. The naming convention for the inference script is <model>_infer_improve.py. Refer to documentation for detailed description:
https://github.com/JDACS4C-IMPROVE/LGBM/blob/develop/lgbm_infer_improve.py

Model

Trained Model:
- `model.json`

Inference (code snippets)

Essential Imports:
```
# Core improvelib imports
import improvelib.utils as frm
# Application-specific (DRP) imports
from improvelib.applications.drug_response_prediction.config import (
DRPInferConfig)
# Model-specifc imports
from model_params_def import infer_params
import lightgbm as lgb
```

Data Loading:
```
# model-specific
test_data = pd.read_parquet(Path(params["input_dir"]) / test_data_fname)
```

Model Loading:
```
model.load_model(str(modelpath)) # model-specific
```

Save Predictions and Scores:
- `frm.store_predictions_df(…)`
- `infer_scores = frm.compute_performance_scores(…)`

Predictions

Test Predictions:
- `test_y_data_predicted.csv`

Test Scores:
- `test_scores.csv`

Other:
- `param_log_file.txt`
- `runtime_infer.json`

LLM utility tool assisting with model standardization

The `improve_prompts` tool generates task-specific prompts users can paste into an LLM to evaluate model repositories for conformance to the IMPROVE code structure. It guides consistent checks before and after curation, covering environment setup, preprocessing, training, required files, stage scripts, and README quality, and expects evidence-based, PASS/FAIL reports. This tool does not replace testing or documentation; rather, it accelerates first-pass curation and helps catch common compliance errors.
improve_prompts repo: https://github.com/JDACS4C-IMPROVE/improve_prompts/tree/master

Supplementary Material S8

Scatter plots of cross-dataset performance scores in y-axis vs. drug-set coverage (left plots) and cell-line coverage (left plots) in the x-axis.

Model	Cross-dataset performance vs Drug-set coverage (Spearman's rank correlation coefficient)	Cross-dataset performance vs Cell-line coverage (Spearman's rank correlation coefficient)
DeepCDR	0.88	0.80
DeepTTC	0.85	0.78
GraphDRP	0.91	0.75
HiDRA	0.84	0.82
LGBM	0.95	0.83
tCNNS	0.86	0.75

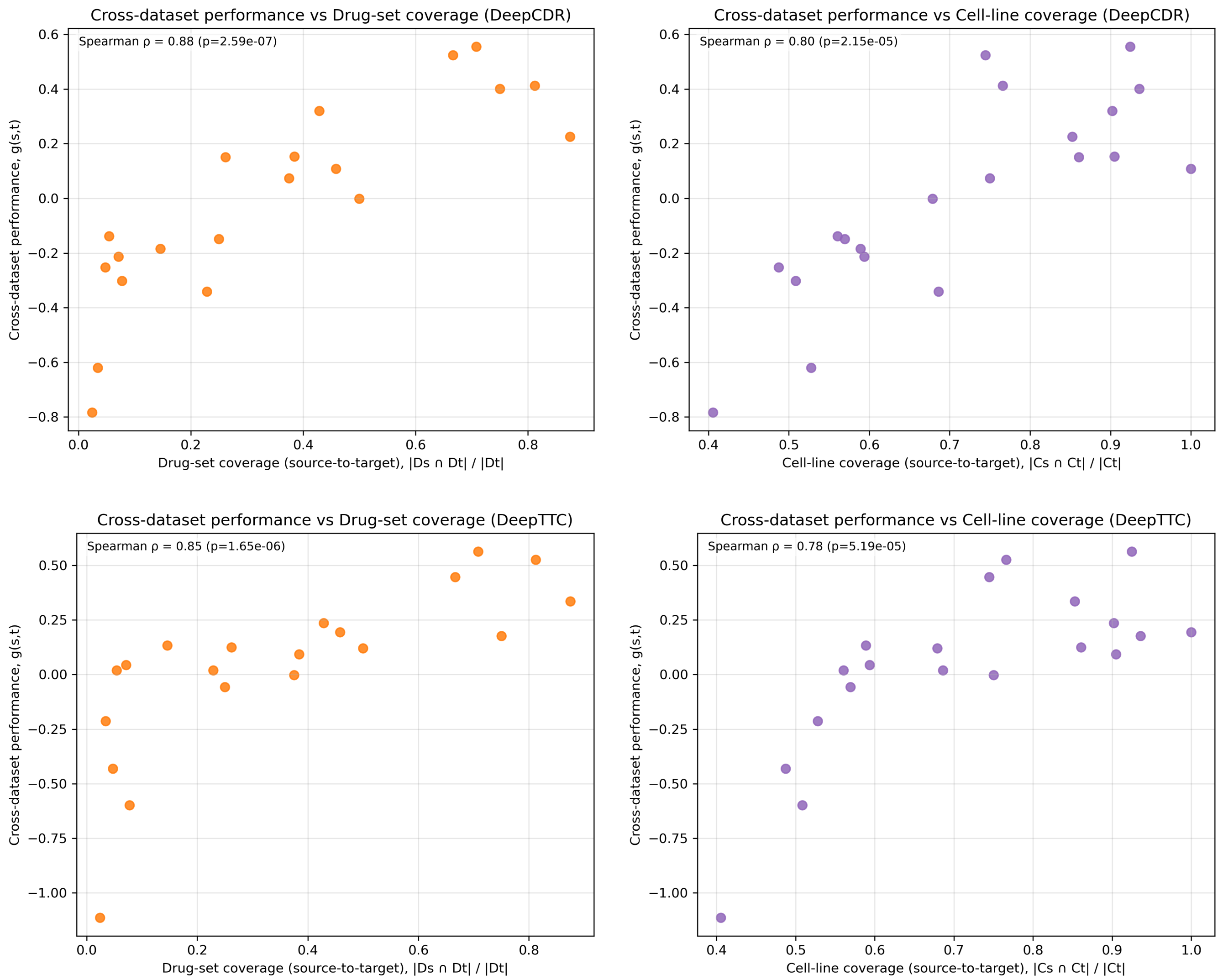

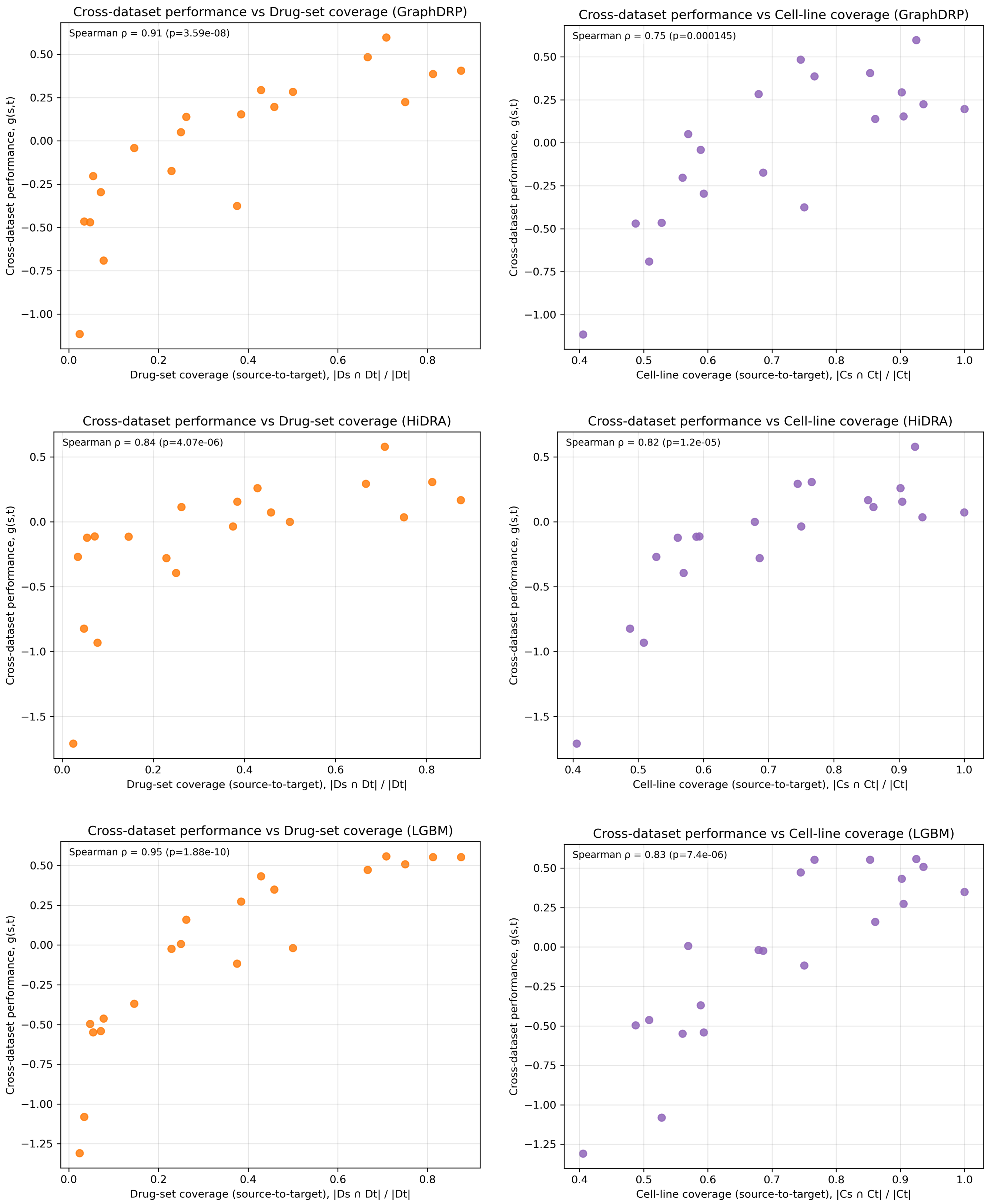

Cross-dataset performance vs Drug-set coverage (GraphDRP)
Spearman ρ = 0.91 (p=3.59e-08)
Cross-dataset performance, g(s,t)
Drug-set coverage (source-to-target), |Ds ∩ Dt| / |Dt|
Cross-dataset performance vs Cell-line coverage (GraphDRP)
Spearman ρ = 0.75 (p=0.000145)
Cross-dataset performance, g(s,t)
Cell-line coverage (source-to-target), |Cs ∩ Ct| / |Ct|
Cross-dataset performance vs Drug-set coverage (HiDRA)
Spearman ρ = 0.84 (p=4.07e-06)
Cross-dataset performance, g(s,t)
Drug-set coverage (source-to-target), |Ds ∩ Dt| / |Dt|
Cross-dataset performance vs Cell-line coverage (HiDRA)
Spearman ρ = 0.82 (p=1.2e-05)
Cross-dataset performance, g(s,t)
Cell-line coverage (source-to-target), |Cs ∩ Ct| / |Ct|
Cross-dataset performance vs Drug-set coverage (LGBM)
Spearman ρ = 0.95 (p=1.88e-10)
Cross-dataset performance, g(s,t)
Drug-set coverage (source-to-target), |Ds ∩ Dt| / |Dt|
Cross-dataset performance vs Cell-line coverage (LGBM)
Spearman ρ = 0.83 (p=7.4e-06)
Cross-dataset performance, g(s,t)
Cell-line coverage (source-to-target), |Cs ∩ Ct| / |Ct|

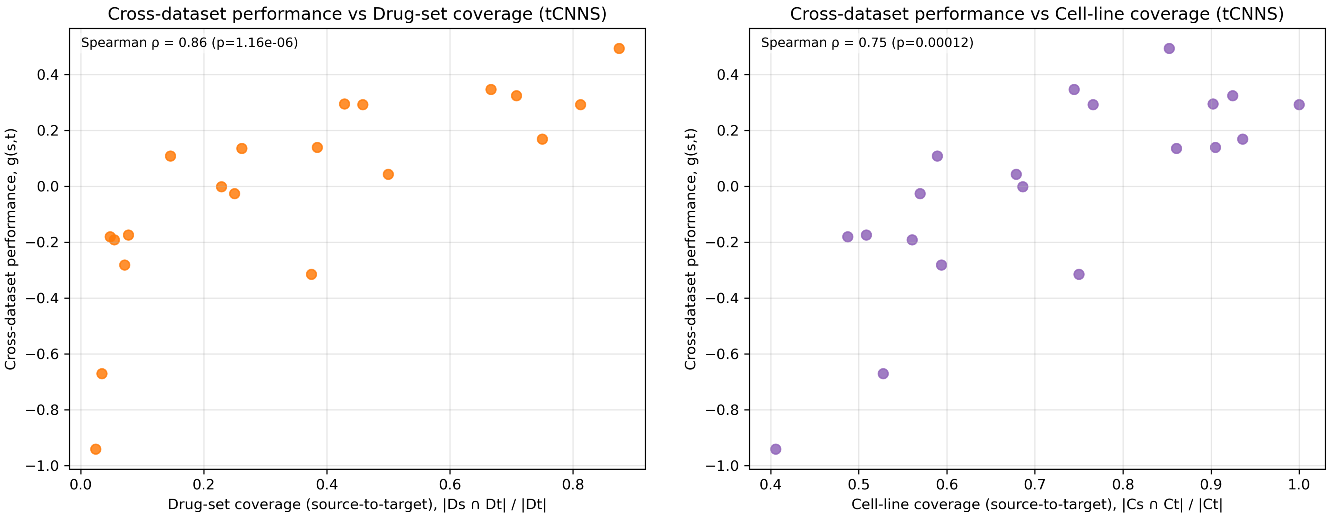

Cross-dataset performance vs Drug-set coverage (tCNNS)
Spearman ρ = 0.86 (p=1.16e-06)
Cross-dataset performance, g(s,t)
Drug-set coverage (source-to-target), |Ds ∩ Dt| / |Dt|
Cross-dataset performance vs Cell-line coverage (tCNNS)
Spearman ρ = 0.75 (p=0.00012)
Cross-dataset performance, g(s,t)
Cell-line coverage (source-to-target), |Cs ∩ Ct| / |Ct|

Supplementary Material S9

Table S9. A model summary of capabilities and plausible limitations in the context of cross-dataset generalization performance. **E = E**mpirical. Observed in our experiments. **L = L**iterature. Supported by prior literature. **H = H**ypothesis/plausible (not tested in this study). Claims refer to our cross-dataset setup; we avoid causal attributions to architecture without controlled experiments (e.g., ablation studies).

**Model**	**Featurization and Architectures**	**Capabilities (E/L/H)**	**Plausible limitations under cross-dataset use (E/L/H)**
DeepCDR	Multimodal with drug molecular graphs (GNN) + 3 types of omics features	Learns cross-modal interactions (L); allows modality ablations (L)	Moderate cross-dataset scores vs top performers (E); may need to learn from smaller datasets when not all omics types are available (H)
DeepTTC	Transformer-based drug encoder (FPs) + gene expressions	Scales with data (H)	Moderate cross-dataset performance vs top performers (E)
GraphDRP	Drug molecular graphs (GNN) + omics features (CNN)	Learns topological drug structure from graphs (L); fast training in our setup (E)	Since the model efficiently learns from molecular drug structures, it may substantially rely on compound overlap (H)
HiDRA	Hierarchical attention over drug and cell representations	Offers attention-based interpretability (L)	Moderate cross-dataset performance vs top performers (E)
LGBM	Tree ensembles (fingerprints + cell features)	Strong baseline with low-to-moderate data sizes (E); feature importance (L)	Underperforms on larger datasets (GDSCv1, CTRPv2) relative to deep learning models (E)
tCNNS	CNN-based sequence encoders (drugs and cells)	Simple architecture and fast training in our setup (E)	Significantly lower performance than other models in our setting (E)
UNO	Multimodal integration of drugs + cell features	Strong cross-dataset performance (E, L); allows modality ablations (L)	Earlier generation model design may have less long-term scaling potential than transformers (H)
